# RadSed-INT: A Scenario-Aware Protocol for Radioactivity Assessment in Dynamic Beach Sediments

**DOI:** 10.3390/toxics14070590

**Published:** 2026-07-03

**Authors:** Sebastiano Ettore Spoto, Roberta Somma, Antonio Trifirò

**Affiliations:** 1 Dipartimento di Scienze della Terra, Università degli Studi di Firenze, 50121 Florence, Italy; 2 Dipartimento di Scienze Matematiche e Informatiche, Scienze Fisiche e Scienze della Terra, Università degli Studi di Messina, 98166 Messina, Italy; antonio.trifiro@unime.it; 3 Istituto Nazionale di Fisica Nucleare, Sezione di Catania, 95123 Catania, Italy

**Keywords:** beach sediments, environmental radioactivity, HPGe gamma-ray spectrometry, grain-size partitioning, heavy-mineral fraction, vertical radiostratigraphy, dust resuspension, inhalation exposure, dose assessment, NORM

## Abstract

Beach sediments may contain natural radionuclides, fallout-derived radionuclides, naturally occurring radioactive material (NORM) or technologically enhanced naturally occurring radioactive material (TENORM), but their radiological significance depends on sediment dynamics and exposure scenario as much as on bulk activity concentration. RadSed-INT is introduced as a tiered field–laboratory protocol for assessing radioactivity in dynamic beach sediments. The protocol links in situ gamma screening, statistically designed transects, primary/confirmatory A/B sampling, high-purity germanium (HPGe) gamma-ray spectrometry, grain-size and heavy-mineral partitioning, vertical mini-core radiostratigraphy, and triggered dust, radon/thoron and ingestion pathway modules. Its central requirement is radiometric mass closure between directly measured bulk activity and the mass-weighted reconstruction from sediment fractions. External and internal doses are evaluated only for explicitly defined material status, exposure pathways, occupancy assumptions and regulatory domains; construction-material and NORM indices are retained as context-specific comparators, not universal beach-sediment limits. RadSed-INT defines escalation triggers from reconnaissance to confirmatory spatial, grain-size, vertical or aerosol investigations. This article is methodological and does not report new primary field data. Extended notation, a date-sensitive regulatory matrix, confounder checklists, implementation tables and a minimal worked example are included to support transparent and reproducible application of the core protocol logic.

## 1. Introduction

Beach sediments are often screened for natural and artificial radioactivity, but the meaning of a measured activity concentration is not self-evident. A value reported in Bq kg^−1^ may represent well-mixed surface sediment, a diluted heavy-mineral lamina, a buried enriched layer that is temporarily hidden below the active beachface, or sediment reworked by storms, nourishment, grooming, erosion or aeolian deflation. These cases are not equivalent for human exposure, nor for regulatory interpretation. Nevertheless, many studies still combine a small number of bulk samples with conventional radiological indices and then draw broad conclusions about environmental safety [1,2,3,4,5].

To address this gap, RadSed-INT is proposed as a tiered field–laboratory protocol that links exploratory in situ screening, statistically designed transects, paired primary/confirmatory A/B sampling, laboratory high-purity germanium (HPGe) gamma-spectrometry confirmation, grain-size partitioning, vertical radiostratigraphy and dose-based decision criteria. The protocol is intended to help assessors decide when a beach-sediment radioactivity survey is sufficient, when it should be repeated in time, and when it should be escalated to detailed spatial, sedimentological or vertical investigation. It is not intended to replace national regulations; rather, it makes explicit the sampling, metrological and scenario assumptions required for their defensible application. The core contribution is the radiometric mass-closure test between directly measured bulk activity and the mass-weighted reconstruction from grain-size fractions. Field screening, vertical profiles, dust screening and regulatory translation are organized around that test as either core controls or trigger-based modules.

For the purposes of this article, the toxicity-relevant endpoint is radiological exposure rather than chemical toxicity. External gamma dose, committed effective dose from inhalation or ingestion, and radon/thoron-related internal exposure are treated as pathway-specific radiological endpoints. The chemical toxicity of uranium, thorium, trace metals, or rare-earth-bearing minerals lies outside the scope of RadSed-INT unless additional geochemical bioavailability and toxicological models are explicitly added. This boundary is important for a toxicology-oriented readership: the protocol concerns toxicological relevance through ionizing-radiation dose, while mineralogical and geochemical measurements are used to explain carrier phases, mobility and sampling representativeness [6,7,8].

A large literature has measured ^226^Ra, ^232^Th and ^40^K in beach sediments by gamma-ray spectrometry and has derived absorbed dose rate, annual effective dose (often reported as annual effective dose equivalent, AEDE), radium equivalent activity and external or internal hazard indices [1,2,3,4,9,10,11,12,13,14]. These studies are valuable because they document high-background areas and relate radioactivity to provenance, heavy-mineral assemblages and coastal hydrodynamics. The limitation arises when the reported quantities are interpreted outside their domain. A dry-mass bulk activity concentration is not independent of drying, sieving, splitting and counting geometry; it is the mass-weighted outcome of texture, density, mineralogy and the analyzed fraction. In the same way, an index derived from ^226^Ra, ^232^Th and ^40^K does not by itself constitute a regulatory threshold for beach sediment left in situ. It may serve as a construction-product screen, an environmental comparator or a historical convention, depending on the context in which it is used [15,16,17,18,19].

This distinction is operationally important because it determines the relevant exposure pathways, the dose quantity to be compared and the applicable regulatory framework. Depending on material status and use, beach sediment may be assessed as part of an open recreational environment, as dredged material to be moved, as a sand resource for nourishment or construction, as naturally occurring radioactive material (NORM) or technologically enhanced NORM (TENORM), or as a site potentially affected by artificial radionuclides. Each case changes the relevant exposure pathway, the dose constraint or reference level, and the regulatory meaning of the same measured activity concentration [6,8,20,21,22]. Sediment with elevated ^232^Th carried by monazite in a natural swash deposit is not equivalent, from a regulatory standpoint, to the same sand sold as an aggregate, stored in a processing facility, disposed as a residue, or contaminated by ^137^Cs after a nuclear accident. In some studies, however, the transition from bulk activity to regulatory interpretation is made without fully defining this chain of interpretation.

The second weakness is metrological. In high-resolution gamma-ray spectrometry, the activity concentration estimate is derived from net peak area, photon emission probability per decay, dry mass, efficiency calibration, matrix density, sample height, coincidence summing, background subtraction, decay corrections and self-attenuation, especially for low-energy photons such as the 46.5 keV emission of ^210^Pb [23,24,25,26]. Counting time does not change the physical activity concentration of the sample; it affects counting statistics, standard uncertainty and detection capability under the adopted measurement procedure. In beach sediments, where carbonate fragments, quartz, Fe-Ti oxides and zircon-rich fractions may have different densities and attenuation properties, the assumption that all fractions are metrologically interchangeable is fragile. A protocol that measures the bulk and the fractions must, therefore, demonstrate closure: the activity concentration of the bulk sample should agree, within combined uncertainty, with the mass-weighted activity concentration reconstructed from its fractions.

A third weak point in many surveys is sedimentological and stratigraphic. A beach surface is only the visible boundary of a mobile sediment body. Thin heavy-mineral laminae may be exposed, buried and re-exposed by storms, swash sorting, aeolian winnowing and mechanical beach management; if the enriched material includes a fine, dry and wind-remobilizable component, external exposure is no longer the only relevant pathway. Grain size is not simply a descriptive accessory. It is a state variable controlling mineral sorting and adsorption surface area. Fine fractions can concentrate radionuclides through clays, organic matter and Fe-Mn oxyhydroxides; fine to medium sand can also concentrate dense resistant accessory minerals and coarse bioclastic or quartz-rich fractions may dilute the radioactivity. The relation between activity concentration and grain size is radionuclide-specific and site-specific; it should, therefore, be tested as a partitioning problem rather than assumed a priori. The observed bulk signal is a constrained mixture [27,28,29,30,31].

A further exposure pathway becomes relevant only under specific conditions: aeolian resuspension and inhalation of radionuclide-bearing dust. In this context, the relevant mechanism is physical aerosolization rather than chemical volatility; U–Th-bearing minerals do not become airborne because they evaporate. Dry, fine, or mechanically disturbed sediment can be lifted, saltating sand can liberate finer particles, and a respirable fraction may then carry natural or artificial radionuclides into the air. International NORM guidance treats airborne dust and, in Th-rich settings, thoron progeny as potentially relevant internal-exposure pathways during dry handling or processing of mineral materials [32,33]. For an open recreational beach this pathway is normally secondary to external gamma exposure, but it may become decision-relevant for dry backshore/dune sediment, grooming, scraping, stockpiling, nourishment works, mineral-sand deposits, windy beaches, or post-event reworking. Accordingly, the aeolian module is triggered only when sediment state and exposure conditions make airborne particles plausible.

In this setting, RadSed-INT is proposed as a grain-size-resolved and scenario-aware protocol for environmental radioactivity in beach sediments. The manuscript does not add another site-specific concentration table to the literature. It formalizes the measurand, makes the grain-size–radioactivity relation testable, and constrains the use of radiological indices to their valid decision domains. The framework is intended to be internationally adaptable: its regulatory module can be re-parameterized for Europe and for other national systems, while leaving final interpretation to current competent-authority guidance. The present article is methodological rather than site-specific: it defines the analytical and mathematical structure into which case-study data can be inserted and does not report new field data. Implementation-oriented, date-sensitive and checklist-like details are provided in the Supplementary Materials: Supplementary Table S1 gives the extended notation, Supplementary Table S2 gives the extended regulatory translation matrix, Supplementary Table S3 gives the extended confounder checklist, Supplementary Table S4 gives HPGe correction and QA requirements, Supplementary Table S5 gives closure-uncertainty schemes, Supplementary Table S6 gives the S4 artificial-radionuclide workflow and Supplementary Table S7 gives Tier 4/Tier 5 trigger indicators, Supplementary Table S8 gives standard sedimentological equations and Supplementary Table S9 gives a minimal bulk–fraction closure worked example.

### 1.1. Why Beach-Sediment Radioactivity Needs a Radiogeochemical Protocol

#### 1.1.1. The Beach as a Heterogeneous Radiogeochemical System

A beach sediment sample is a physical mixture. At minimum it contains mineral grains with different density, size, shape and provenance. In many coastal settings it also contains shell fragments, lithic grains, organic debris, anthropogenic particles and transient heavy-mineral laminae. If $x_{h}$ denotes the horizontal position on the beach surface, ζ denotes sampling depth, $d_{g}$ denotes particle diameter, and μ denotes mineral or particle class, the activity concentration of radionuclide or decay-chain segment *j* measured in the laboratory may be written conceptually as

(1) $$C_{j}^{P,G}\left(x_{h},\zeta \right)=M\left[S(x_{h},\zeta ,d_{g},\mu ),P,G\right],$$

where S is the sediment state, P is the preparation operator and G is the counting geometry. In transect-based applications, $x_{h}$ is reduced to the cross-shore coordinate, whereas alongshore variability is represented by replicated transects or by explicit alongshore stratification; no alongshore uniformity is assumed unless it is declared. Equation (1) is deliberately formal. It states that activity concentration is not only a property of the field material; it is also the result of the preparation and measurement operations by which the field material is converted into a radiometric sample. A bulk activity reported as “beach sand, $\mathrm{Bq}\;{\mathrm{kg}}^{-1}$” is, therefore, incomplete unless P and G are specified.

This is especially important for beaches enriched in heavy minerals. Zircon can host U and Th through lattice substitution and radiation damage; monazite is commonly enriched in Th and rare earth elements (REE); allanite and titanite may carry U–Th–REE signatures; Fe-Ti oxides can control magnetic and density fractions and carbonate bioclasts may dilute the activity of siliciclastic heavy-mineral assemblages [30,31,34,35]. The field pattern can change over meters or even centimeters after storms. A single composite bulk sample cannot reveal whether an elevated activity is produced by a spatially extensive geochemical background, by a thin heavy-mineral veneer, by a fine fraction, or by an anthropogenic particle population.

#### 1.1.2. Common Radiological Indices and Their Domain of Validity

Environmental radioactivity studies often report radium equivalent activity $Ra_{eq}$, external hazard index $H_{ex}$, internal hazard index $H_{in}$ and activity concentration index *I*. These indices are useful for comparison, but they are not interchangeable with site-specific dose assessment. The activity concentration index used in European radiological protection of building materials,

(2) $$I=\frac{C_{Ra}}{300}+\frac{C_{Th}}{200}+\frac{C_{K}}{3000},$$

where activity concentrations are in $\mathrm{Bq}\;{\mathrm{kg}}^{-1}$, is a screening tool for gamma radiation emitted by building materials, not a criterion for beach sediment left in situ [16,17,18]. The same caution applies to Russian-style effective specific activity indices for construction materials and to national building-material standards such as GB 6566 in China [18,36,37]. These quantities may be relevant if beach sand becomes a product or a construction aggregate. They are not, by themselves, evidence that a recreational beach exceeds a regulatory dose criterion.

The protocol proposed here retains conventional indices as optional comparability metrics, but separates them from the regulatory decision. That decision begins with the scenario: Is the sediment left in situ? Is it dredged, relocated, or stockpiled? Is it used as a material? Is artificial contamination present? Only after this scenario has been specified can the appropriate dose criterion, reference level, exemption concept, or product index be selected. Table 1 summarizes the intended decision domain of the most common indices and the corresponding interpretive limitation that the present protocol is designed to control.

#### 1.1.3. From Threshold Calculation to Scenario-Aware Interpretation

The methodological weakness that motivates RadSed-INT is not the calculation of conventional indices. It is the conversion of those indices into regulatory conclusions without preserving the domain in which they were defined. Let $Q(C_{Ra},C_{Th},C_{K})$ be any radiological quantity derived from measured activity concentrations. A defensible decision requires at least four additional operators:

(3) $$D=R\left[Q\left(C_{Ra},C_{Th},C_{K}\right),\;S_{mat},\;E_{path},\;O,\;J\right],$$

where $S_{mat}$ is the status of the material, $E_{path}$ is the exposure pathway, O is the occupancy or use scenario, and J is the applicable jurisdictional framework. Omitting these terms changes the problem. Sediment left in situ is not a building product; a stockpiled dredged sand is not a recreational surface; a naturally enriched heavy-mineral lamina is not necessarily an accident-derived contaminated waste and a post-accident radiocesium criterion is not a generic NORM threshold. The same numerical value of $C_{j}$ can, therefore, lead to different decisions because the regulated object has changed.

A published dataset illustrates this limitation without requiring a hypothetical case. In a beach-sediment study from the Ionian coast of Calabria, Caridi et al. reported for site ID5 $C_{Ra}=60.2\pm 6.4$ Bq kg^−1^, $C_{Th}=524\pm 73$ Bq kg^−1^ and $C_{K}=148\pm 20$ Bq kg^−1^ [4]. The same site was associated with a calculated absorbed dose rate of about $351\;\mathrm{nGy}\;h^{-1}$ and an annual effective dose, reported in the original study as annual effective dose equivalent (AEDE), of 106μSv a^−1^ for a beach occupancy of 432 h a^−1^. If the three activity concentrations are instead inserted into the European building-material index, Equation (2) gives

(4) $$I=\frac{60.2}{300}+\frac{524}{200}+\frac{148}{3000}\approx 2.87.\;$$

This value would be relevant if the sediment were evaluated as a building material or construction aggregate. It does not, by itself, mean that the beach sediment left in situ exceeds a regulatory limit for recreational use. Conversely, the annual effective dose calculated for a summer tourist scenario cannot certify acceptability for construction use, stockpiling, dusty handling, worker exposure, or post-storm remobilization. With the same dose rate, a high-occupancy maintenance scenario of 2000 h a^−1^ would correspond to approximately $0.49\;\mathrm{mSv}\;a^{-1}$, which is again a different decision object. The example is used here in a constructive sense. It does not question the published measurements or the formulas themselves. It highlights a scenario-transfer weakness that can arise when thresholds are moved from one domain to another without redefining material status, exposure pathway, occupancy and jurisdiction. RadSed-INT is designed to make that transfer explicit before any regulatory interpretation [6,8,16,17,20,38].

**Figure 1:**
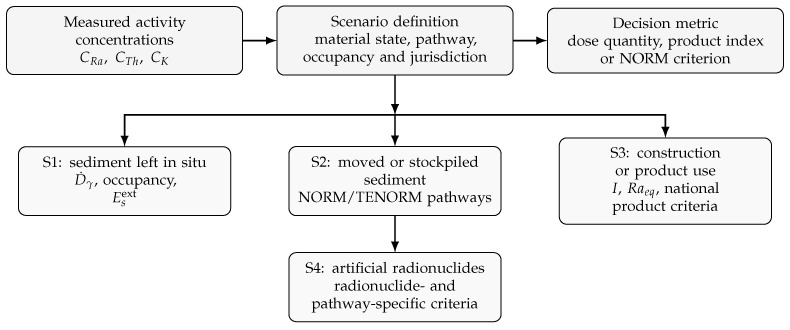
Scenario-transfer limitation and the role of scenario definition. The same measured activity concentrations can support different decisions only after the material state, exposure pathway, occupancy and jurisdictional context have been specified.

Figure 1 summarizes the same scenario-transfer logic as a decision sequence rather than as a direct threshold comparison.

## 2. Materials and Methods

Throughout this paper, notation is kept deliberately conservative because several physical quantities have similar conventional symbols. In particular, the letter A is not used for activity concentration because the protocol also uses A/B to identify primary and retained confirmatory samples. Throughout the manuscript, A denotes the primary analytical sample and B denotes the retained confirmatory sample; an A/B pair denotes that paired design and is not used as a synonym for ordinary laboratory split duplicates. The term retained confirmatory sample is used where chain-of-custody or evidentiary retention is specifically discussed. Table 2 summarizes the notation used in the equations and decision rules.

The Supplementary Material is an integral supporting file for implementing the protocol, not an independent dataset. The main manuscript retains the core decision logic, minimum notation and reporting requirements. Supplementary Table S1 provides the extended notation, Supplementary Table S2 provides the extended multi-jurisdictional regulatory translation matrix, Supplementary Table S3 provides the full environmental and methodological confounder checklist, Supplementary Table S4 provides HPGe correction and quality-assurance items, Supplementary Table S5 provides alternative closure-uncertainty propagation schemes, Supplementary Table S6 provides an S4 operation tree for artificial radionuclides and Supplementary Table S7 provides quantitative trigger indicators for vertical and aeolian escalation. These supplementary tables are cited at the points where the corresponding main-text elements are introduced, and they should be updated, where relevant, at the time of case-study application.

### 2.1. Core Protocol and Trigger-Based Modules

RadSed-INT is organized as a core protocol plus diagnostic modules. The core protocol contains the minimum elements needed for a defensible beach-sediment radioactivity assessment: scenario definition, stratified field screening, paired primary/confirmatory A/B sampling where confirmation is needed, dry-mass HPGe measurement, characteristic limits, grain-size mass balance and scenario-specific external-dose translation. Additional modules are activated only by explicit triggers such as field–laboratory mismatch, visible heavy-mineral laminae, sediment relocation, dust generation, artificial radionuclides, or management decisions. This hierarchy is used throughout the manuscript to prevent ordinary reconnaissance studies from being burdened with every specialist test, while preserving a clear escalation route for heterogeneous or high-stakes cases. The resulting core tiers and trigger-based modules are summarized in Table 3.

For routine monitoring laboratories, the minimum viable implementation is intentionally narrower than the full protocol. It consists of a documented scenario definition, a reproducible field-screening design, dry-mass HPGe gamma-ray spectrometry of representative bulk samples, characteristic limits, a declared external-dose scenario and a decision on whether grain-size closure, vertical radiostratigraphy or dust/aerosol modules are triggered. Where the equipment or budget does not allow a full Tier 3–5 implementation, the report should state which modules were not activated, why they were not required, and which uncertainty or representativeness limitations remain. This fallback structure is intended to facilitate adoption by routine laboratories without weakening the distinction between screening and confirmatory assessment.

### 2.2. Mathematical and Metrological Framework

#### 2.2.1. Activity Concentration from Gamma-Ray Spectrometry

For a gamma line *ℓ* of radionuclide or decay-chain segment *j*, the dry-mass activity concentration is estimated as

(5) $${\hat{C}}_{j\ell }=\frac{N_{\ell }^{net}}{t_{c}\;m_{\mathrm{dry}}\;\varepsilon_{\ell }\left(G,\rho_{s},H_{\mathrm{fill}}\right)\;P_{\gamma ,\ell }}\;F_{\ell }^{att}\;F_{\ell }^{sum}\;F_{\ell }^{dec}\;F_{\ell }^{geo},$$

Here $N_{\ell }^{net}$ is the background-subtracted net peak area, $t_{c}$ is live counting time and $m_{\mathrm{dry}}$ is dry mass. The term $\varepsilon_{\ell }$ is the full-energy peak efficiency for geometry G, sediment matrix density $\rho_{s}$ and fill height $H_{\mathrm{fill}}$; $P_{\gamma ,\ell }$ is the photon emission probability per decay. The *F* terms correct for self-attenuation, coincidence summing, decay or ingrowth, and residual geometry mismatch. Each $F_{q,\ell }$ is defined so that the corrected activity estimate equals the uncorrected estimate multiplied by $F_{q,\ell }$. Correction factors are applied only for effects not already included in the net peak area or in the efficiency calibration. If gross peak areas are used instead, background subtraction and its uncertainty must be written explicitly, for example $N_{\ell }^{net}=N_{\ell }^{gross}-B_{\ell }$, and must not be duplicated as a multiplicative background factor. The equation is standard in gamma-ray spectrometry, but its explicit use is central here because each term can change when a beach sample is split into fractions with different density and matrix composition [23,24,26,39,40]. For implementation, the efficiency and correction model shall be documented for each matrix class actually counted. Quartz-rich, carbonate-rich and heavy-mineral-rich fractions can differ in density, fill height and attenuation; they should, therefore, be counted in calibrated geometries or corrected through validated efficiency-transfer, transmission, Monte Carlo or matrix-matched approaches. Low-energy peaks, especially ^210^Pb at 46.5 keV, require matrix-specific self-attenuation correction or thickness–density calibration curves. Coincidence-summing corrections are required only when the source–detector geometry and cascade structure make them relevant; count-rate effects require control of dead time, pile-up and geometry rather than simply shortening counting time. The minimum operational items are listed in Supplementary Table S4.

For radionuclides determined from multiple gamma lines, line-specific estimates should not be averaged arithmetically unless their uncertainties are equal. When estimates share systematic components, such as dry mass, efficiency calibration, geometry transfer, density correction, self-attenuation, or nuclear-data inputs, the generalized estimator is

(6) $${\hat{C}}_{j}=\frac{1^{T}V^{-1}c}{1^{T}V^{-1}1},\;u^{2}\left({\hat{C}}_{j}\right)={\left(1^{T}V^{-1}1\right)}^{-1},$$

where c is the column vector of line-specific estimates, V is their covariance matrix and 1 is a conformable column vector of ones. Throughout the manuscript, u(X) denotes the standard uncertainty of *X*, and $u^{2}\left(X\right)$ denotes the corresponding variance or squared standard uncertainty. If off-diagonal covariance terms are unavailable or demonstrably negligible, Equation (6) reduces to the diagonal inverse-variance approximation

(7) $${\hat{C}}_{j}=\frac{\sum_{\ell =1}^{L_{j}}{\hat{C}}_{j\ell }/u^{2}\left({\hat{C}}_{j\ell }\right)}{\sum_{\ell =1}^{L_{j}}1/u^{2}\left({\hat{C}}_{j\ell }\right)},\;u^{2}\left({\hat{C}}_{j}\right)={\left(\sum_{\ell =1}^{L_{j}}\frac{1}{u^{2}\left({\hat{C}}_{j\ell }\right)}\right)}^{-1}.$$

Equations (7) and (9) are fallback approximations used only when off-diagonal covariance terms are unavailable or demonstrably negligible. A consistency statistic can then be calculated. When the full covariance matrix is available, the appropriate generalized contrast is

(8) $$Q_{j}={\left(c-{\hat{C}}_{j}1\right)}^{T}V^{-1}\left(c-{\hat{C}}_{j}1\right),$$

which can be compared, as a diagnostic approximation, with a $\chi^{2}$ distribution with $L_{j}-1$ degrees of freedom. If only diagonal uncertainties are available, this reduces to the reduced statistic

(9) $$\chi_{\nu }^{2}=\frac{1}{L_{j}-1}\sum_{\ell =1}^{L_{j}}\frac{{\left({\hat{C}}_{j\ell }-{\hat{C}}_{j}\right)}^{2}}{u^{2}\left({\hat{C}}_{j\ell }\right)}.$$

High $Q_{j}$ or $\chi_{\nu }^{2}$ values should trigger review of peak interferences, coincidence summing, efficiency interpolation, self-attenuation, density/geometry mismatch and decay-chain disequilibrium. For ^226^Ra, the usual HPGe determination relies on progeny lines of ^214^Pb and ^214^Bi after sealing the sample to allow ingrowth of radon daughters; for the thorium series, ^228^Ac, ^212^Pb and ^208^Tl lines must be interpreted in relation to ^228^Ra and ^228^Th equilibrium [24,26]. The activity of ^210^Pb at 46.5 keV is not acceptable without a matrix-specific self-absorption correction or equivalent calibration strategy [25].

The standard uncertainty of Equation (5) may be approximated, for independent input quantities, by the logarithmic propagation equation

(10) $$\begin{array}{cc} {\left(\frac{u(C)}{C}\right)}^{2}\approx & {\left(\frac{u(N)}{N}\right)}^{2}+{\left(\frac{u(t_{c})}{t_{c}}\right)}^{2}+{\left(\frac{u(m_{\mathrm{dry}})}{m_{\mathrm{dry}}}\right)}^{2}\\ \; & +{\left(\frac{u(\varepsilon )}{\varepsilon }\right)}^{2}+{\left(\frac{u(P_{\gamma })}{P_{\gamma }}\right)}^{2}+\sum_{q}{\left(\frac{u(F_{q})}{F_{q}}\right)}^{2}. \end{array}$$

In practice, $u(t_{c})$ is negligible relative to counting statistics, efficiency and matrix corrections, but it is retained here for dimensional completeness. Equation (10) is an independent-input approximation; if mass, efficiency, density, geometry or self-attenuation corrections are shared among samples, fractions or gamma lines, the corresponding covariance terms must be included in the uncertainty budget. Decision thresholds and detection limits should be computed with a recognized radiometric framework rather than inferred from arbitrary multiples of background count rate [41,42]. General uncertainty propagation and covariance treatment follow the Guide to the Expression of Uncertainty in Measurement (GUM) framework [43]. In this paper the decision threshold $y^{*}$ denotes the level above which the presence of the measurand is decided, the detection limit $y^{\#}$ denotes the smallest true value expected to be detected with the stated error probabilities, and any laboratory minimum detectable activity (MDA) or reporting limit is treated as an operational, method-specific reporting quantity. The MDA shall, therefore, not be used as a synonym of detection limit unless the laboratory has formally defined it that way in the measurement procedure. Values at or below $y^{*}$ are treated as not decided for presence; values above $y^{*}$ but below $y^{\#}$ are treated as detected but not reliably quantifiable and are not used as quantified inputs to dose, trend or regression calculations unless the validated procedure explicitly supports such use. Equation (10) is intended for quantified peaks sufficiently above the decision threshold; results close to detection limits should be treated within a characteristic-limits framework rather than by relative uncertainty propagation alone. Left-censored observations should be handled with methods appropriate for censored environmental data, such as maximum likelihood, regression on order statistics, or Bayesian hierarchical approaches, rather than by universal substitution of MDA/2 unless that approximation is explicitly justified for a descriptive screening table [44]. Such substitution, if used at all, is limited to explicitly labeled descriptive summaries and is not used for dose decisions, correlations, trend analysis, model fitting, or regulatory interpretation. Operationally, RadSed-INT reports such results as left-censored observations, e.g., <$y^{*}$, <$y^{\#}$ or <MDA with the sample-specific limit used by the laboratory. If a substitution is used only to display an explicitly labeled descriptive table, the rule must be declared in the caption or table note. For modeling, correlation analysis, or trend evaluation, censored-data methods are the default; if censoring is too extensive for stable estimation, the result should be reported qualitatively as non-detected within the achieved detection capability rather than forced into a numerical model.

#### 2.2.2. Secular Equilibrium and What HPGe Actually Measures

A frequent source of interpretive ambiguity is the naming of decay-chain quantities. HPGe spectra do not usually measure the parent nuclide ^238^U directly in a dried beach sand by a single robust line; they estimate chain segments. If a sample is closed to radon and equilibrium has been re-established, ^214^Pb and ^214^Bi can be used as proxies for ^226^Ra. If ^226^Ra is assumed to represent the ^238^U series, that assumption is an additional geochemical statement, not an automatic consequence of gamma-ray spectrometry.

Let $A_{p}$ be the activity of parent *p* and $A_{d}\left(t\right)$ the activity of a daughter *d* with decay constant $\lambda_{d}$ produced from a long-lived parent whose activity is approximately constant over the ingrowth period. If $A_{d}\left(0\right)=0$, then

(11) $$A_{d}\left(t\right)=A_{p}\left(1-e^{-\lambda_{d}t}\right).$$

If the daughter activity is not initially zero, the more general first-order form is $A_{d}\left(t\right)=A_{p}+\left[A_{d}\left(0\right)-A_{p}\right]e^{-\lambda_{d}t}$. For sealed samples used to estimate ^226^Ra from ^214^Pb and ^214^Bi, the operationally dominant ingrowth is that of ^222^Rn. If the dry sample is radon-retentive and ^222^Rn is initially absent or removed during preparation, the ingrowth factor, i.e., the fraction of equilibrium activity reached at sealing time $t_{s}$, is

(12) $$F_{Rn}\left(t_{s}\right)=1-e^{-\lambda_{Rn}t_{s}},\;A_{Rn}\left(t_{s}\right)=A_{Ra}F_{Rn}\left(t_{s}\right),$$

where $t_{s}$ is the time since sealing. The quantity $F_{Rn}$ is a growth fraction, not the correction multiplier for estimating the parent. If daughter activity measured before complete ingrowth is used to infer ^226^Ra, the corresponding correction in Equation (5) is the reciprocal, $1/F_{Rn}\left(t_{s}\right)$. The short-lived daughters ^214^Pb and ^214^Bi equilibrate much faster after radon ingrowth, but they can be used for ^226^Ra only if container tightness, sealing date, headspace, seal type, container material and gamma-line consistency are documented. A waiting period of approximately four weeks is, therefore, a minimum operational rule for sealed samples, and longer periods may be chosen for quality assurance (QA) consistency. The result should be named as ^226^Ra or as a specific chain segment unless independent evidence justifies the notation ^238^U. For the thorium series, reported quantities should distinguish the ^228^Ra–^228^Ac segment from the ^228^Th–^212^Pb–^208^Tl segment whenever secular equilibrium has not been demonstrated. The shorthand ^232^Th should be used only when the relevant thorium-series equilibrium assumptions have been verified or explicitly stated.

#### 2.2.3. Grain-Size-Resolved Mass Balance

Let a dried sediment sample be separated into *G* particle-size fractions. The dry mass of fraction *i* is $m_{i}$, its mass fraction is

(13) $$w_{i}=\frac{m_{i}}{\sum_{k=1}^{G}m_{k}},\;\sum_{i=1}^{G}w_{i}=1,$$

and the activity concentration of radionuclide *j* in that fraction is $C_{ij}$. The next step is a mass-balance prediction. This prediction is valid only if no material is lost and the analyzed bulk belongs to the same population as the generated fractions. The closure equation applies to one exhaustive partition at a time: particle-size fractions must be mutually exclusive and collectively recover the counted bulk mass. Heavy-mineral, magnetic or density concentrates are diagnostic subfractions nested within a parent size class unless the full hierarchical mass partition is reconstructed. Under these conditions, the predicted bulk activity concentration is

(14) $$C_{bj}^{pred}=\sum_{i=1}^{G}w_{i}C_{ij}.$$

The measured bulk activity concentration $C_{bj}^{meas}$ must then satisfy

(15) $$Z_{j}=\frac{C_{bj}^{meas}-C_{bj}^{pred}}{\sqrt{u^{2}\left(C_{bj}^{meas}\right)+u^{2}\left(C_{bj}^{pred}\right)-2\;\mathrm{cov}\left(C_{bj}^{meas},C_{bj}^{pred}\right)}}$$

with $|Z_{j}|≲2$ for approximate 95% consistency if normal uncertainty assumptions are acceptable. If the bulk and fractions are measured in independent preparations, the covariance term is often negligible. If the same calibration, mass-balance variables, or grain-size mass fractions $w_{i}$ are shared, covariance should not be ignored; the $w_{i}$ are measured compositional quantities constrained by $\sum_{i}w_{i}=1$. In compact form, the uncertainty of the predicted bulk can be written as

(16) $$u^{2}\left(C_{bj}^{pred}\right)={\left(\nabla_{x}C_{bj}^{pred}\right)}^{T}\;\Sigma_{x}\;\left(\nabla_{x}C_{bj}^{pred}\right),$$

where $x={\left(w_{1},\ldots ,w_{G},C_{1j},\ldots ,C_{Gj}\right)}^{T}$ and $\Sigma_{x}$ contains both analytical and mass-fraction covariance terms. This closure test detects loss of fines, poor homogenization, density-related efficiency bias, self-attenuation bias, fraction contamination and mismatch between the counted bulk and the later fractionated material. It is not applied by summing ordinary grain-size classes and diagnostic heavy-mineral concentrates at the same hierarchical level unless the mass balance has been reconstructed for the entire nested split. Supplementary Table S5 summarizes two practical propagation schemes. The independent scheme is used only when bulk and fractions have separate mass, geometry and efficiency inputs. The shared-parameter scheme retains covariance from common efficiency calibration, dry-mass normalization, sieving recovery, density correction, or common radionuclide nuclear data. Neglecting shared covariance can bias the magnitude of $Z_{j}$, either by over-stating or under-stating the closure discrepancy depending on the sign of the covariance term.

Two derived quantities are useful:

(17) $$EF_{ij}=\frac{C_{ij}}{C_{bj}^{meas}},$$

which describes the radiometric enrichment of fraction *i*, and

(18) $$RC_{ij}=\frac{w_{i}C_{ij}}{C_{bj}^{pred}},\;\sum_{i}RC_{ij}=1,$$

which describes the fractional contribution of size class *i* to the bulk activity of radionuclide *j*. This distinction matters. A size fraction may have a high enrichment factor but a low bulk contribution if its mass fraction is small. Conversely, a moderately enriched but abundant fraction may dominate the bulk activity concentration, and therefore, the dose-rate estimate. Enrichment factors, contribution ratios, vertical ratios and log-scale contrasts should be reported only for radionuclides or decay-chain segments that are quantitatively resolved; they should not be calculated from left-censored values or from denominator terms below the adopted quantification capability.

#### 2.2.4. Mineral-Carrier Model

The size-fraction activity can be decomposed into mineral carriers and surface-associated components:

(19) $$C_{ij}=\sum_{\mu =1}^{M}q_{i\mu }c_{\mu j}+C_{ij}^{surf}+C_{ij}^{art},$$

where $q_{i\mu }$ is the mass fraction of mineral carrier μ in grain-size class *i*, $c_{\mu j}$ is the carrier-specific activity concentration, $C_{ij}^{surf}$ is activity concentration associated with adsorbed or authigenic phases, and $C_{ij}^{art}$ is the activity concentration of artificial radionuclides in that fraction. For ^226^Ra, ^232^Th and ^40^K, the first term can dominate where resistant heavy minerals are enriched; for ^137^Cs and total ^210^Pb, the surface-associated term can be important in fine fractions. In this protocol, ^210^Pb denotes total ^210^Pb unless a supported–unsupported separation has been explicitly carried out; the term excess or unsupported ^210^Pb should be used only when that component has been operationally defined by subtraction or modeling. Equation (19) is also a useful warning: the relation between activity concentration and grain size is radionuclide-specific and site-specific. It should be tested as a partitioning problem rather than assumed a priori.

When chemical data are available, elemental concentrations can be converted to activities for cross-checking. For a radionuclide *r* with half-life $T_{1/2,r}$, atomic mass $M_{r}$ and decay constant $\lambda_{r}=\ln2/T_{1/2,r}$, the activity concentration in a 1 kg sample containing mass fraction $x_{r}$ of the radionuclide of interest is

(20) $$C_{r}=x_{r}\frac{N_{A}}{M_{r}}\lambda_{r},$$

where $N_{A}$ is Avogadro’s constant and $M_{r}$ is expressed in $\mathrm{kg}\;{\mathrm{mol}}^{-1}$. In Equation (20), the half-life $T_{1/2,r}$ must be expressed in seconds when calculating activity concentration in Bq kg^−1^. If geochemical data are reported as elemental concentrations, $x_{r}$ must be obtained by multiplying the elemental mass fraction by the appropriate isotopic abundance where needed. The ppm and percentage values in the following approximations are expressed on a mass basis; the derivation is given in Appendix A. For natural geochemical reporting, the approximate conversions are

(21) $$\begin{array}{cc} 1\;\mathrm{ppm}\;U\;\mathrm{by}\;\mathrm{mass} & ≃12.35\;\mathrm{Bq}\;{\mathrm{kg}}^{-1}\;{(}^{238}U),\\ 1\;\mathrm{ppm}\;\mathrm{Th}\;\mathrm{by}\;\mathrm{mass} & ≃4.06\;\mathrm{Bq}\;{\mathrm{kg}}^{-1}\;{(}^{232}\mathrm{Th}),\\ 1\;\mathrm{wt}.\%\;K & ≃313\;\mathrm{Bq}\;{\mathrm{kg}}^{-1}\;{(}^{40}K). \end{array}$$

These conversions are useful for coherence checks between high-purity germanium gamma-ray spectrometry (HPGe), X-ray fluorescence (XRF) and inductively coupled plasma mass spectrometry (ICP-MS), but they do not replace direct gamma-ray spectrometric determination of the radiological measurand. In particular, an elemental U concentration does not prove equilibrium with ^226^Ra in a weathered sediment, nor does a Th concentration prove equilibrium among all thorium-chain daughters.

#### 2.2.5. Absorbed Dose Rate and Scenario-Specific Effective Dose

For external terrestrial gamma exposure, the absorbed dose rate in air at 1 m above ground is commonly estimated from the activity concentrations of ^226^Ra, ^232^Th and ^40^K by

(22) $${\dot{D}}_{\gamma }=0.462\;C_{Ra}+0.604\;C_{Th}+0.0417\;C_{K},$$

where ${\dot{D}}_{\gamma }$ is in $\mathrm{nGy}\;h^{-1}$ and $C_{Ra}$, $C_{Th}$ and $C_{K}$ are in $\mathrm{Bq}\;{\mathrm{kg}}^{-1}$ [38]. Equation (22) is retained as a screening approximation for a laterally homogeneous, semi-infinite source geometry at 1 m above ground. The coefficients are model-based conversion factors for soil-like media and should be treated as screening coefficients unless supported by field dose-rate measurements or site-specific Monte Carlo calculations. In disequilibrium cases, $C_{Ra}$ and $C_{Th}$ must denote the decay-chain segment responsible for the gamma field, not an unverified parent radionuclide. For beach sediments, this is particularly important where high-activity material occurs as thin heavy-mineral veneers, buried laminae, discrete particles or strong vertical/lateral gradients rather than as a semi-infinite homogeneous half-space. Where contact dose rate, 1 m field dose rate and HPGe-derived screening dose do not agree within their stated uncertainty and support, Equation (22) remains only a screening estimate; direct in situ dose-rate measurements, Tier 4 depth profiles or site-specific transport modeling should control the final interpretation.

For an exposure scenario *s* with occupancy $T_{s}$ in hours per year, the annual external effective dose can be reported either as a site-related quantity or as an incremental quantity:

(23) $$E_{s,site}^{\mathrm{ext}}={\dot{D}}_{\gamma ,site}\;T_{s}\;0.7\times 10^{-6},\;\Delta E_{s}^{\mathrm{ext}}=\left({\dot{D}}_{\gamma ,site}-{\dot{D}}_{\gamma ,bg}\right)\;T_{s}\;0.7\times 10^{-6}.$$

Both quantities are in $\mathrm{mSv}\;a^{-1}$ when dose rates are in $\mathrm{nGy}\;h^{-1}$. The factor 0.7 Sv Gy^−1^ is the conventional conversion from absorbed dose in air to effective dose for environmental external exposure [38]. The first term describes the scenario dose associated with the measured site sediment; the second is used only when a local/regional background or pre-intervention baseline has been explicitly defined and subtracted. The background term ${\dot{D}}_{\gamma ,bg}$ must be measured or justified for comparable geology, geomorphic zone, season, moisture state and measurement height; otherwise, the assessment should report a site-related dose rather than an incremental dose. Any regulatory comparison must state which of these quantities is being compared and whether the decision concerns the sediment left in situ, sediment relocation, stockpiling, handling, reuse, or contamination. Equation (23) remains a screening relation and does not replace a full radiological assessment where inhalation of dust, radon/thoron exhalation, ingestion, occupational exposure, or artificial radionuclides are relevant. When respirable or inhalable dust is a plausible pathway, the external-dose calculation must be complemented by the specialist aeolian module defined in Section 2.3.9.

A second internal pathway, usually minor for ordinary recreational use but relevant in child exposure, contaminated fine sediment, dusty handling, or occupational contact, is incidental ingestion. A minimal screening expression is

(24) $$E_{s}^{\mathrm{ing}}=10^{3}\;M_{s}^{ing}\sum_{j}C_{j,ing}\;e_{j}^{ing},$$

where $E_{s}^{\mathrm{ing}}$ is in mSv a^−1^ if $M_{s}^{ing}$ is the annual mass of sediment incidentally ingested in scenario *s* in kg a^−1^, $C_{j,ing}$ is the activity concentration of radionuclide *j* in the ingestible fraction in Bq kg^−1^, and $e_{j}^{ing}$ is the ingestion dose coefficient in Sv Bq^−1^. The factor $10^{3}$ converts Sv to mSv. Equation (24) should not be applied by default to every beach. It is a triggered screening pathway for cases where sediment contact is plausible and the relevant ingestible material can be defined. The exposure factor and dose coefficient must be scenario-specific, including the relevant age class and radionuclide form where such distinctions are provided. Example scenarios include child beach play, maintenance work, stockpile handling or contaminated fine sediment; assumptions should be based on documented soil/sediment ingestion factors rather than on arbitrary values [7,45,46].

Pathway-aware Sediment Dose Index forms are, therefore, defined rather than a single universal index. The total pathway dose is defined only when the internal pathways have been triggered and quantified:

(25) $$\begin{array}{cc} E_{s}^{\mathrm{tot}} & =E_{s}^{\mathrm{ext}}+E_{s,ann}^{\mathrm{inh}}+E_{s}^{\mathrm{ing}}+E_{s}^{\mathrm{Rn}/\mathrm{Tn}},\\ SDI_{s,\mathrm{ext}} & =\frac{E_{s}^{\mathrm{ext}}}{E_{ref,s}},\;SDI_{s,\mathrm{tot}}=\frac{E_{s}^{\mathrm{tot}}}{E_{ref,s}},\;\Delta SDI_{s}=\frac{\Delta E_{s}^{\mathrm{ext}}}{E_{ref,s}}. \end{array}$$

For external-dose screening alone, $E_{s}^{\mathrm{ext}}$ in $SDI_{s,\mathrm{ext}}$ is a placeholder for the declared external-dose quantity and must be stated explicitly as either the site-related term $E_{s,site}^{\mathrm{ext}}$ or the incremental term $\Delta E_{s}^{\mathrm{ext}}$ from Equation (23). The equivalent dose-rate reference is

(26) $${\dot{D}}_{ref,s}=\frac{E_{ref,s}}{0.7\times 10^{-6}\;T_{s}}.$$

The total-pathway form $SDI_{s,\mathrm{tot}}$ is reported only when internal pathways have been explicitly triggered and quantified; otherwise, pathway-specific doses are reported separately. Unlike $Ra_{eq}$ or *I*, the scenario-specific SDI forms contain occupancy and a declared comparison level. A recreational tourist, a resident who spends many hours on a beach, and a worker who handles dry sand are not represented by the same index. The SDI family is an internal protocol descriptor for transparent screening and prioritization; it is not presented as a regulatory index recognized by a competent authority.

The choice of $E_{ref,s}$ is not a mathematical default but a declared decision variable. In this protocol, $E_{ref,s}$ is a declared comparison value, not a universal beach limit, and is never implicit. Before interpreting any SDI form, the assessor shall define whether the comparison is made against total site-related dose, incremental dose above local or regional background, or incremental dose associated with sediment relocation, handling, or reuse. The selected $E_{ref,s}$ shall, therefore, be reported together with the exposure scenario, the representative individual, the occupancy basis, the included pathways and the regulatory or screening framework used. Table 4 gives the operational selection logic used in RadSed-INT.

Table 5 translates Equation (26) into illustrative dose-rate references for selected occupancy assumptions.

### 2.3. Statistical and Environmental Design

#### 2.3.1. The Statistical Population Must Be Stated Before Samples Are Counted

The question “how many samples are needed?” has no physically meaningful answer until the population, spatial support and decision objective have been defined. A sample collected from the upper 5 cm of a dry berm is not a replicate of a sample collected from the active swash zone, and neither is it interchangeable with a laboratory split taken from the same bag. RadSed-INT, therefore, treats sampling as a design problem rather than as a fixed number. The design must state whether the target is (i) an average activity concentration for a sedimentary unit, (ii) the upper tail of a heterogeneous beach, (iii) a comparison between morphodynamic zones, (iv) a temporal trend, (v) the detection of small high-activity patches, or (vi) the estimation of dose to a defined user group.

This requirement follows the same logic as general environmental data-quality planning: the required type, number and location of observations must be linked to the intended decision or estimate, and the tolerable uncertainty and decision tolerance must be stated before the sampling plan is fixed [47,48,49,50]. In beach radiogeochemistry, the principle is especially important because the most radiologically enriched material may occupy a small spatial support: a thin black-sand lamina, a storm lag, a swash-line concentration, or an anthropogenically reworked patch. A dataset composed of many laboratory replicates from a single composite may, therefore, look precise while still being spatially unrepresentative.

The operational population is defined here as a homogeneous coastal-sedimentary unit during a specified geomorphic state. Within that unit, the basic sampling coordinates are transect *t*, cross-shore zone *z*, depth interval *h*, campaign time *q*, field replicate *r* and radionuclide *j*. The measurand may be the activity concentration $C_{j}$ of a bulk sediment, the activity concentration of a grain-size fraction, a dose-rate quantity, or a derived index such as $SDI_{s,\mathrm{ext}}$.

#### 2.3.2. Tiered Escalation and Temporal Confirmation

RadSed-INT is not a single fixed sampling recipe. It is a decision architecture. Tier 1 is an exploratory investigation designed to learn the beach: its radiological range, cross-shore structure, obvious anomalies, accessibility, sedimentological units and immediate field dose-rate pattern. A Tier 1 dataset may justify a statement such as “no anomaly was detected under the surveyed conditions”, but it should not by itself support a definitive regulatory conclusion for a dynamic or managed beach. A stronger statement requires either temporal confirmation under comparable conditions or escalation to a confirmatory tier.

The transition from Tier 1 to higher tiers is governed by explicit triggers rather than by subjective concern. For a field quantity *X* measured at station *i*, for example, contact dose rate, 1 m dose rate, count rate, or a preliminary activity estimate, a robust local anomaly score may be defined as

(27) $$Z_{i}^{rob}=\frac{g\left(X_{i}\right)-\mathrm{median}\left\{g\left(X_{b}\right)\right\}}{1.4826\;\mathrm{MAD}\{g\left(X_{b}\right)\}},$$

where $X_{b}$ denotes values from the same coastal-sedimentary background stratum, *g* is either the identity or logarithmic transform, and MAD is the median absolute deviation. Values of $|Z^{rob}|\geq 3$ are treated as operational anomalies, and values $|Z^{rob}|\geq 3.5$ are treated as strong anomalies unless a documented instrument or environmental artifact explains them. If uncertainties are available for two values $X_{1}$ and $X_{2}$, the uncertainty-normalized contrast is

(28) $$Z_{12}=\frac{X_{1}-X_{2}}{\sqrt{u^{2}\left(X_{1}\right)+u^{2}\left(X_{2}\right)-2\;\mathrm{cov}\left(X_{1},X_{2}\right)}}.$$

The covariance term should be retained when shared calibration, geometry, background, or field conditions are relevant; the diagonal form is a fallback approximation when shared components are unavailable or demonstrably negligible. A contrast is not promoted to an anomaly merely because it is visually striking; it must be reproducible, statistically resolvable, or physically explained by sedimentary evidence such as a heavy-mineral lamina.

**Figure 2:**
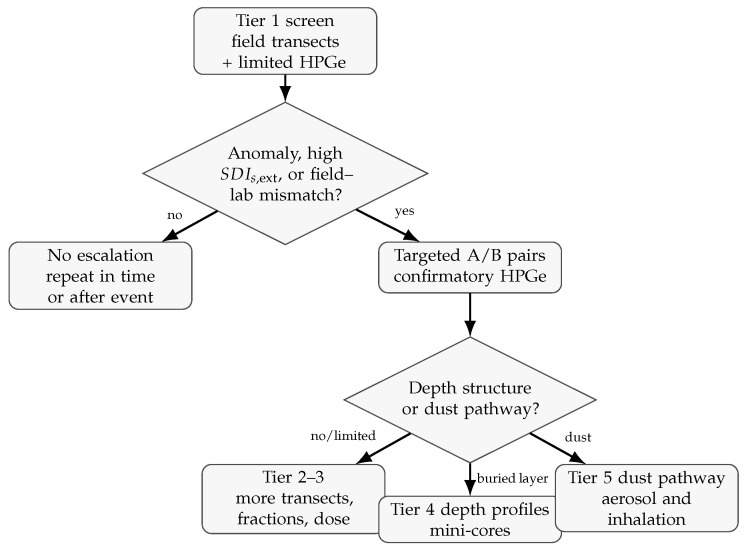
Escalation logic of RadSed-INT. Tier 1 is exploratory. Absence of escalation does not mean that the site is closed permanently; temporal repetition or post-event verification remains part of baseline maintenance where beach dynamics justify it. Tier 1 becomes confirmatory only after temporal repetition or targeted A/B analysis of retained confirmatory samples. Tier 4 is activated when depth structure controls exposure or sediment management; Tier 5 is activated only when dry fine material may become respirable dust.

The decision logic is summarized in Figure 2. If Tier 1 shows no robust anomaly, no field–laboratory inconsistency and $SDI_{s,\mathrm{ext}}$ remains in the green screening class, the recommended action is temporal confirmation rather than immediate escalation. Low-energy, unmanaged beaches may be rechecked every three to five years or after major geomorphic change. Dynamic, touristic, nourished, or mechanically groomed beaches should be rechecked annually to biennially, and always after nourishment, dredging, exceptional storms, overwash, excavation, or visible black-sand formation. If a Tier 1 anomaly is observed, the protocol moves to targeted Tier 2 or Tier 3 sampling, with paired primary/confirmatory A/B samples and HPGe confirmation. If the anomaly may be vertically displaced, Tier 4 vertical radiostratigraphy is activated. If the concern is dry, wind-erodible, or mechanically disturbed sediment that may generate respirable dust, the optional Tier 5 aeolian resuspension and inhalation module is activated as a specialist pathway tier.

The escalation triggers used in the protocol are listed in Table 6. They are deliberately conservative because the cost of a false negative is not only radiological: it is also interpretive. A thin enriched unit missed by a shallow or poorly timed sample can later be exposed by wave erosion, scraped by beach maintenance, or incorporated into a nourishment stockpile.

#### 2.3.3. Hierarchical Variance Model

For statistical analysis, activity concentrations are commonly better represented on a logarithmic scale because beach radioactivity data are often right-skewed when heavy minerals occur in patches. Let

(29) $$Y_{jctzhqr}=g\left(C_{jctzhqr}\right),\;g\left(C\right)=\log\left(C\right)\;\mathrm{or}\;g\left(C\right)=C,$$

where *c* is the coastal-sedimentary unit, *t* is the transect, *z* is the cross-shore zone, *h* is the depth interval, *q* is the sampling campaign and *r* is the field replicate. A general mixed model for the protocol is

(30) $$Y_{jctzhqr}=\mu_{j}+\alpha_{cj}+\tau_{ctj}+\gamma_{zj}+\delta_{hj}+\theta_{qj}+{\left(\gamma \theta \right)}_{zqj}+x_{ctzhq}^{T}\beta_{j}+\epsilon_{jctzhqr},$$

where α, τ, γ, δ and θ represent coastal-unit, transect, cross-shore, depth and campaign effects. The vector x contains measured covariates such as median grain size $D_{50}$, sorting, fine fraction $F_{<63}$, heavy-mineral mass fraction $f_{HM}$, carbonate or shell fraction, moisture at collection, recent wave energy, distance from river mouth, and indicators of nourishment or grooming. Some terms may be fixed effects and others random effects, depending on the inference. The residual term can be decomposed as

(31) $$\epsilon_{jctzhqr}=\epsilon_{jctzhqr}^{field}+\epsilon_{jctzhqr}^{split}+\epsilon_{jctzhqr}^{lab}+\epsilon_{jctzhqr}^{count},$$

so that field heterogeneity is not confused with counting uncertainty. This distinction is essential: a long HPGe count can reduce $\epsilon^{count}$ but cannot repair an under-sampled swash deposit. When the response variable includes observations below $y^{*}$, $y^{\#}$, or a laboratory MDA/reporting limit, these models must not be fitted after naive substitution such as MDA/2; censored-data methods or an explicitly restricted quantified subset are required.

For each radionuclide, a pilot campaign should estimate variance components:

(32) $$\sigma_{tot,j}^{2}=\sigma_{unit,j}^{2}+\sigma_{transect,j}^{2}+\sigma_{zone,j}^{2}+\sigma_{depth,j}^{2}+\sigma_{time,j}^{2}+\sigma_{field,j}^{2}+\sigma_{lab,j}^{2}.$$

The final sampling density is then based on the largest components. If most variance is between cross-shore zones, more transects with the same zonation are efficient. If variance is dominated by localized hot patches, a grid or adaptive design is needed. If variance is dominated by time, repeated campaigns are mandatory.

#### 2.3.4. Sample Size for Estimating a Baseline Mean

For a homogeneous stratum and approximately independent observations, a first-order sample-size requirement for estimating a mean with a two-sided confidence interval of relative half-width *r* is

(33) $$n_{eff}\geq {\left(\frac{z_{1-\alpha /2}\;CV}{r}\right)}^{2},$$

where $CV=s/\bar{C}$ is the coefficient of variation estimated from a pilot survey and $z_{1-\alpha /2}$ is the standard normal quantile. Equation (33) is not a universal rule; it is a transparent planning equation. For skewed data, the same calculation should be performed on the log scale:

(34) $$\sigma_{L}=\sqrt{\ln(1+CV^{2})},\;n_{eff}\geq {\left(\frac{z_{1-\alpha /2}\;\sigma_{L}}{\ln(1+r)}\right)}^{2}.$$

If samples are clustered within transects, the effective size must be corrected by the design effect (DEFF)

(35) $$DEFF=1+\left(m_{c}-1\right)\rho_{c},\;n_{raw}=n_{eff}\;DEFF,$$

where $m_{c}$ is the average number of observations per cluster and $\rho_{c}$ is the intraclass correlation. Consequently, many samples from a few transects can be less informative than fewer samples distributed across more independent transects.

Table 7 gives the resulting independent sample sizes for typical coefficients of variation and target precisions.

The implication for beach sediments is direct. A low-heterogeneity carbonate or quartz beach may be characterized at the screening level with about 10–20 independent surface samples per homogeneous unit. A heavy-mineral beach with CV near 0.5 requires roughly 25 independent samples for a 20% relative confidence half-width. Highly patchy black-sand systems can require many more observations unless the design explicitly stratifies the enriched patches.

#### 2.3.5. Sample Size for Differences, Trends and Hot-Spot Detection

If the aim is to detect a difference Δ between two independent groups, for example, pre- and post-nourishment sediments or high- and low-energy beach sectors, the approximate per-group sample size on the chosen analysis scale is

(36) $$n_{g}\geq \frac{2\;{\left(z_{1-\alpha /2}+z_{1-\beta }\right)}^{2}\;\sigma^{2}}{\Delta^{2}},$$

where 1−β is statistical power. For repeated transects measured before and after an event, the paired design is more efficient:

(37) $$n_{p}\geq \frac{{\left(z_{1-\alpha /2}+z_{1-\beta }\right)}^{2}\;\sigma_{d}^{2}}{\Delta^{2}},$$

where $\sigma_{d}$ is the standard deviation of paired differences. This is the mathematical reason for reoccupying the same transects through time: it removes part of the between-transect variance from the test.

For localized high-activity patches, a different calculation is required. If a patch occupies a fraction *p* of the survey area and *n* independent locations are sampled randomly, the probability of intersecting the patch at least once is

(38) $$P_{det}=1-{\left(1-p\right)}^{n},\;n\geq \frac{\ln(1-P_{det}^{*})}{\ln(1-p)},$$

where $P_{det}^{*}$ is the desired detection probability. To obtain $P_{det}=0.95$, a patch occupying 10% of the area requires about 29 independent locations; a 5% patch requires about 59; a 1% patch requires about 299. This calculation explains why a small number of bulk samples can miss radiologically important black-sand lenses. In practice, RadSed-INT combines systematic or stratified sampling with in situ gamma scanning so that judgmental follow-up samples are triggered by measured anomalies rather than by visual impression alone.

#### 2.3.6. Surface Depth and Vertical Radiostratigraphic Transects

The expression “surface sediment” is often used as if it denoted a point at ζ=0. It does not. A sediment sample has a finite vertical support, and that support is part of the measurand. Let ζ be the depth below the contemporary beach surface, positive downward. The activity concentration of radionuclide or chain segment *j* measured in a depth interval $\left[h_{1},h_{2}\right]$ is the dry-mass-weighted average

(39) $$C_{j}\left[\zeta_{1},\zeta_{2}\right]=\frac{\int_{\zeta_{1}}^{\zeta_{2}}\rho_{d}\left(\zeta \right)C_{j}\left(\zeta \right)\;d\zeta }{\int_{\zeta_{1}}^{\zeta_{2}}\rho_{d}\left(\zeta \right)\;d\zeta },$$

where $\rho_{d}\left(\zeta \right)$ is dry bulk density. If density is approximately constant within the interval, Equation (39) reduces to a thickness-weighted average. The corresponding inventory per unit area is

(40) $$I_{j}\left[\zeta_{1},\zeta_{2}\right]=C_{j}\left[\zeta_{1},\zeta_{2}\right]\int_{\zeta_{1}}^{\zeta_{2}}\rho_{d}\left(\zeta \right)\;d\zeta ,$$

which is useful because a thin surface veneer may have high activity concentration but small inventory, whereas a thicker buried layer may be the more important reservoir if erosion or excavation exposes it.

A simple dilution calculation illustrates the problem. If a radiogenic lamina of thickness δ and activity concentration $C_{\ell }$ is embedded in background sediment of activity concentration $C_{0}$ and homogenized through an interval of thickness $H_{m}$, the measured activity is

(41) $$C_{mix}=C_{0}+\frac{\delta }{H_{m}}\left(C_{\ell }-C_{0}\right).$$

Equation (41) assumes approximately uniform dry bulk density across the mixed interval and complete homogenization of the lamina and background sediment within the support $H_{m}$. Thus, a 1 cm lamina with ten times the background activity produces only a 45% increase if it is homogenized through 20 cm. If the same lamina is isolated as a centimetric increment, it is immediately visible. A protocol based only on a 0–5 cm sample and a broad 5–20 cm sample can, therefore, miss or understate radiologically meaningful stratification.

RadSed-INT, therefore, distinguishes four vertical supports. First, the routine *surface bulk* interval is 0–5 cm. It is recommended for baseline comparability and usually provides enough mass for HPGe spectrometry and grain-size splitting. Second, the exposure veneer interval is 0–2 cm; it is added when visible black-sand films, recent fallout, contact exposure, fresh swash laminae, or contact gamma anomalies are relevant. Third, the routine subsurface check is 5–20 cm; it tests whether enrichment is confined to the present surface or persists beneath it. Fourth, a vertical radiostratigraphic transect is triggered when the depth position of the enriched material can change either exposure or sediment management.

**Figure 3:**
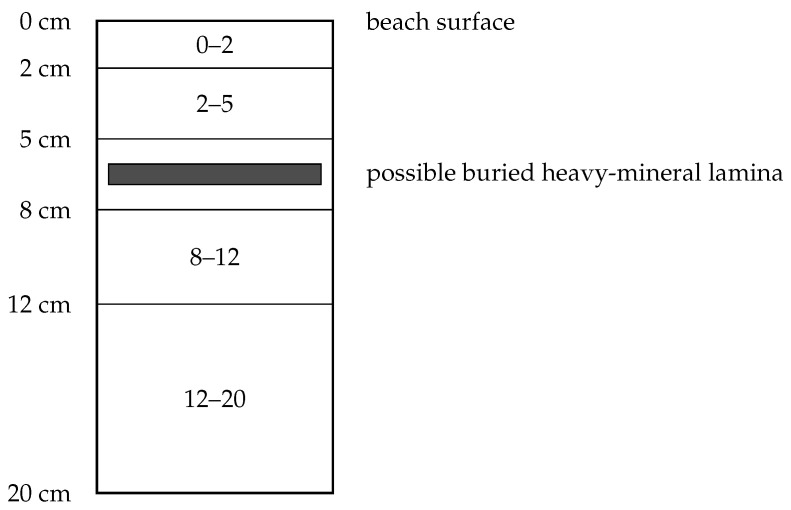
First-resolution mini-core for a triggered vertical radiostratigraphic transect. The shallow 5–8 cm interval is isolated because buried radiogenic laminae can be diluted by broad 5–20 cm composites but can become exposure-relevant after erosion, scraping, or storm reworking.

Figure 3 illustrates how this finite-interval support separates a shallow buried lamina from broader composite intervals. The recommended first-resolution vertical mini-core uses the following finite intervals: 0–2, 2–5, 5–8, 8–12 and 12–20 cm. The 5–8 cm increment is deliberately separated. It is below the ordinary 0–5 cm surface sample, but still shallow enough to be exposed by modest erosion, beach scraping, swash reworking, or storm deflation. If the field question is framed around an “8 cm” level, the sample should not be described as a point sample at 8 cm; it should be collected as a finite interval, for example, 5–8 cm or 8–12 cm, with its support stated explicitly. Where the beach is accretionary, nourished, storm-built or mechanically reworked, the mini-core should be extended to 20–40 cm and, where feasible, 40–60 cm or to the visible sedimentological contact.

Vertical transects are not a routine burden imposed on every beach. They are the Tier 4 diagnostic step of the protocol. They are required when at least one trigger is present. Typical triggers include visible black-sand laminae or scarp layering, contact gamma anomalies, disagreement between contact readings, 1 m dose rate and 0–5 cm HPGe activity, recent storm erosion, overwash or post-storm accretion, nourishment, dredging, scraping or mechanical grooming, a preliminary contrast between 0–5 and 5–20 cm intervals, or a decision about excavation, relocation, reuse or disposal. The scientific question is then no longer only “what is exposed today?” It also becomes “what radiogenic unit can be exposed if the beach surface is lowered by a few centimeters or decimeters?” Beach stratigraphic studies show that surface and shallow subsurface grain size can vary over centimeter-to-decimeter scales. Lower beach and swash-zone sediments may be reworked into layers and patches rather than a uniform column [51,52,53]. Coastal studies of radionuclides and black sands similarly show that activity may vary vertically and that heavy-mineral layers can be radiologically distinct from surrounding sand [34,54,55,56].

For each mini-core increment *k*, RadSed-INT reports the activity ratio

(42) $$VR_{j,k}=\frac{C_{j,k}}{C_{j}\left[0,5\right]},$$

and the activity-depth centroid

(43) $${\bar{\zeta }}_{j}=\frac{\sum_{k}{\bar{\zeta }}_{k}m_{k}C_{j,k}}{\sum_{k}m_{k}C_{j,k}},$$

where ${\bar{\zeta }}_{k}$ is the midpoint depth and $m_{k}$ is dry mass. A surface veneer is indicated when the maximum activity is in 0–2 cm and decreases downward. A buried radiogenic unit is indicated when the maximum activity is below 5 cm, especially in 5–8 or 8–12 cm. A vertically uniform profile is indicated when adjacent increments are indistinguishable after uncertainty propagation. Pairwise vertical differences can be tested on the log scale using

(44) $$Z_{j,k\ell }=\frac{\ln C_{j,k}-\ln C_{j,\ell }}{\sqrt{u^{2}\left[\ln C_{j,k}\right]+u^{2}\left[\ln C_{j,\ell }\right]-2r_{k\ell }u\left[\ln C_{j,k}\right]u\left[\ln C_{j,\ell }\right]}},$$

where $r_{k\ell }$ is the correlation coefficient between uncertainty components shared by the two intervals. If shared components are not propagated explicitly, setting $r_{k\ell }=0$ is conservative for most field contrasts. A contrast with $|Z_{j,k\ell }|>1.96$ is treated as significant at approximately 95% confidence, subject to the usual caution about multiple comparisons.

The depth profile also changes the interpretation of in situ gamma measurements. To first order, the contribution of an areally extensive layer at depth ζ to a gamma signal at photon energy *E* is attenuated by the overlying dry-mass thickness,

(45) $$d\Phi_{E}\left(\zeta \right)\propto C_{j}\left(\zeta \right)\rho_{d}\left(\zeta \right)\exp\left[-{\left(\mu /\rho \right)}_{E}\int_{0}^{\zeta }\rho_{d}\left(s\right)\;ds\right]d\zeta ,$$

where ${\left(\mu /\rho \right)}_{E}$ is the sediment mass attenuation coefficient and the integral is the overlying dry mass per unit area. For constant dry density, the exponential term reduces to $\exp[-{\left(\mu /\rho \right)}_{E}\rho_{d}\zeta ]$. Equation (45) is only a first-order kernel and should not be used in place of Monte Carlo dose modeling or calibrated in situ gamma-ray spectrometry. It explains, however, why contact measurements, 1 m dose rates and laboratory depth increments may respond differently to the same buried layer. Guidance and methodological work on in situ gamma-ray spectrometry have long recognized that unknown depth distribution is a major source of interpretation uncertainty [57,58,59,60].

The practical output of the vertical tier is a classification: surface veneer, buried radiogenic layer, vertically mixed background, storm-laminated sequence or anthropogenically disturbed fill. This classification directly affects management. A surface veneer controls immediate contact exposure and wind redistribution. A buried layer may produce little surface expression under stable accretion but can be re-exposed by storms, excavation, or beach scraping. A vertically uniform profile supports treating the measured bulk as a persistent sedimentary background. Without this distinction, the same measured Bq kg^−1^ value can be assigned the wrong environmental meaning.

For future case studies, Tier 4 escalation can be operationalized with investigation triggers rather than universal risk thresholds. A buried increment is flagged when its vertical ratio $VR_{j,k}$ is greater than 2 and the log-scale contrast in Equation (44) is statistically resolved; $VR_{j,k}$ greater than 5 is treated as a strong enrichment trigger. These values are protocol triggers, not regulatory thresholds, and may be adjusted for local background variability. Exposure after erosion can be expressed as

(46) $$P_{\mathrm{exh},k}\left(\tau \right)=P\left[D_{e}\left(\tau \right)\geq \zeta_{top,k}\right],$$

where $D_{e}\left(\tau \right)$ is the event- or period-specific erosion, scraping, or excavation depth and $\zeta_{top,k}$ is the depth to the top of layer *k*. When local storm, grooming, or erosion-depth distributions are unavailable, Equation (46) is used qualitatively and the assumptions must be stated. Supplementary Table S7 lists the corresponding Tier 4 and Tier 5 trigger indicators.

#### 2.3.7. Recommended Minimum Designs

The protocol uses the tiered design in Table 8. The numbers are not regulatory requirements; they are defensible starting points that must be adjusted after the pilot variance assessment. All counts refer to independent field samples per homogeneous coastal-sedimentary unit, not laboratory repeats from the same bag. Tier 1 is exploratory; Tiers 2 and 3 are confirmatory; Tier 4 is a triggered vertical diagnostic tier; Tier 5 is a triggered aerosolizable-fraction and inhalation-pathway tier.

A future empirical implementation intended to validate or apply the protocol should normally meet Tier 2 for at least one study area. Tier 3 should be applied to any sector where black sands, high dose rates, or management decisions are present. Tier 4 is activated when the vertical position of the enriched unit can change exposure or sediment management. Tier 5 is reserved for cases in which dry dust generation or inhalation may be a non-negligible pathway. Tier 1 remains appropriate as reconnaissance, as a preliminary map used to design the full study, or as a low-risk screen only when followed by temporal confirmation.

#### 2.3.8. Timing: Weather, Tide, Season and Repeated Transects

Laboratory activity concentrations are reported on a dry-mass basis; therefore, the weather at the moment of sampling does not change the decay rate of ^226^Ra, ^232^Th or ^40^K. Weather does, however, change the sediment population being sampled and the field radiation signal. Rain, waves and wind can move fine particles, expose or bury heavy-mineral laminae, alter moisture and packing density, and change the gamma attenuation of the upper sediment. For in situ gamma measurements, rainfall can also produce a short-term increase in ambient gamma dose rate because radon progeny such as ^214^Pb and ^214^Bi are scavenged by precipitation and deposited near the ground surface [61]. Thus, field dose-rate measurements made during or immediately after rain are not directly comparable with fair-weather surveys unless the objective is specifically to characterize rainy conditions.

The default timing rules are, therefore, as follows. First, the baseline in situ survey should be performed during stable meteorological conditions, preferably after at least 48–72 h without significant rainfall, overwash or storm-wave reworking. Second, all cross-shore zones should be sampled within the same tidal window, ideally around low tide for intertidal access, while recording tidal stage, beach elevation and the position of the active swash. Third, repeated campaigns should be anchored both to fixed coordinates and to morphological coordinates such as berm crest, high-tide strandline, dry recreational beach, beach face and swash zone. A fixed point may migrate from one morphodynamic zone to another after erosion; a morphological point may shift horizontally. Both pieces of information are needed.

Repeated transects are required whenever the inference concerns a baseline intended to survive time, a comparison between seasons, a beach-nourishment or dredging action, or a high-energy coast. The recommended minimum is two campaigns: a low-energy or tourist-season campaign and a high-energy or post-winter-storm campaign. A stronger first-year design uses four campaigns. Event-based sampling should be added when significant storms occur, for example within 24–72 h after a storm whose significant wave height exceeds a local high percentile, after documented overwash, or after visible formation of black-sand layers; a recovery survey two to four weeks later should then test whether the anomaly is persistent, transient, buried or re-exposed. Where event sampling follows erosion or accretion, at least a subset of Tier 4 vertical mini-cores should be repeated. Natural radionuclides have been used as tracers of coastal sediment transport and erosion/accumulation processes, and recent work explicitly shows that repeated periods and sediment characteristics are central to their interpretation [51,62].

#### 2.3.9. Tier 5: Aerosolizable Fraction, Wind Resuspension and Inhalation Pathway

In this context, the relevant mechanism is physical aerosolization rather than chemical volatility. Radionuclide-bearing fines, clay aggregates, salt-crusted particles, or heavy-mineral dust can be deflated, resuspended and inhaled. Tier 5 is, therefore, a specialist tier for the inhalation pathway. It is activated only when the field situation makes airborne sediment plausible: dry fine-rich backshore or dune sediment, strong winds, salt-crust breakdown, mechanical beach grooming, vehicle traffic, dredged or nourished sand stockpiles, visible dust during handling, artificial radionuclides associated with fine particles, or strong enrichment of radionuclides in sediment fines that have been linked to an airborne particle-size convention. The sedimentological <63μm fraction must not be used as a surrogate for PM_10_, inhalable, thoracic or respirable aerosol unless a validated aerosolization, dustiness or size-selection method links the sediment fraction to the airborne particle-size convention used for dose assessment. Grain-size classes used for sedimentology and health-related aerosol classes answer different questions and must be reported separately when Tier 5 is activated.

Tier 5 has three aims. First, it tests whether the aerosolizable fraction is radiologically different from the bulk 0–5 cm sediment. Second, it separates wind-driven resuspension from handling-driven dustiness, because a natural dry beach, a stockpile and a mechanically groomed surface do not have the same emission mechanism. Third, it prevents the inhalation pathway from being invoked qualitatively without measurements. A defensible Tier 5 record should report surface moisture, recent wind and rainfall, morphodynamic zone, visible crusting or saltation, fine fraction, dust-generation or aerosol-sampling method, particle-size convention, airborne mass concentration or dustiness mass fraction, and $C_{x,j}$ for the emitted or size-selected fraction.

For scenario *s*, a screening inhalation dose may be written as

(47) $$E_{s,ann}^{\mathrm{inh}}=10^{3}\;T_{s}\;BR_{s}\sum_{j}\sum_{x}C_{j,x}^{air}\;e_{j,x}^{inh},$$

Here $E_{s,ann}^{\mathrm{inh}}$ is in mSv a^−1^ if $T_{s}$ is annual occupancy in h a^−1^. The breathing rate is $BR_{s}$ in m^3^ h^−1^, and $C_{j,x}^{air}$ is the air activity concentration of radionuclide *j* in particle class *x* in Bq m^−3^. The selected particle-size convention, such as inhalable, thoracic, respirable, PM_10_ or a measured aerosol class, must be declared once for the scenario and used consistently for airborne mass concentration, sediment fraction activity and dose coefficients. The dose coefficient $e_{j,x}^{inh}$ is selected consistently with age class, particle-size convention and absorption type, and is expressed in Sv Bq^−1^. The factor $10^{3}$ converts Sv to mSv. If $T_{s}$ describes a short handling or wind event rather than annual occupancy, the result is an event dose and shall be annualized before comparison with annual external-dose metrics or any SDI form. If the airborne material is represented by health-related size fraction *x*, then

(48) $$C_{j,x}^{air}=M_{x}C_{x,j},$$

with $M_{x}$ in kg m^−3^ and $C_{x,j}$ in Bq kg^−1^. If several health-related particle classes are characterized simultaneously, $C_{j}^{air}$ must be reported either as class-specific $C_{j,x}^{air}$ values or as an explicitly stated sum over *x*, with the dose-coefficient basis declared. If a surface-inventory approach is used instead, the equivalent screening form is

(49) $$C_{j}^{air}=K_{R}\;I_{j,res},$$

where $I_{j,res}$ is the resuspendable surface inventory. If $I_{j,res}$ is expressed in Bq m^−2^ and $C_{j}^{air}$ in Bq m^−3^, then $K_{R}$ has units of m^−1^. Equations (47)–(49) are not generic beach-dust models; they define the minimum quantities that must be measured or conservatively bounded before inhalation is used in a radiological decision. Particle-size conventions should follow health-related aerosol definitions, and intake coefficients should follow respiratory-tract and radionuclide-dose-coefficient guidance [7,63,64,65]. When both wind deflation and human disturbance are plausible, the inhalation term should be decomposed as $E_{s,ann}^{\mathrm{inh}}=E_{s,wind}^{\mathrm{inh}}+E_{s,work}^{\mathrm{inh}}$, with separate airborne mass terms, durations and particle-size conventions for natural wind events and beach maintenance, dredging, grooming or stockpile handling. Tier 5 is activated by measurable indicators such as visible dust, dry erodible surface conditions, documented dust-generating operations, airborne PM or inhalable dust above local background, or enrichment of the aerosolizable fraction relative to the bulk. Supplementary Table S7 provides a minimum trigger checklist.

A practical sequence is as follows: (i) measure $F_{<63}$ as a sedimentological fine fraction and, where possible, the <10μm or laser-derived fine tail, without treating them as PM_10_ unless aerosol size selection has been performed; (ii) determine whether radionuclides are enriched in these fractions relative to the bulk; (iii) for stockpiles, dry nourished sand or mechanically disturbed sediment, use field aerosol sampling or a reproducible dustiness test; (iv) estimate Equation (47) only if dust loading, particle-size convention, breathing rate, occupancy and dose coefficients are declared. This structure is consistent with radionuclide-resuspension assessment, NORM heavy-mineral-sand experience and dust-generation practice, all of which show that airborne activity is strongly site- and disturbance-specific [33,66,67,68,69,70,71,72,73]. In most wet, coarse or undisturbed beaches Tier 5 will not be needed. In dry, fine-rich, managed or contaminated settings, it can be decisive because the most radiologically relevant material may be the small mass fraction that becomes airborne rather than the visually dominant bulk sand.

#### 2.3.10. Third-Order Controls and Confounding Effects

A defensible protocol must report the factors that can influence either the sediment activity itself or the measurement of it. Table 9 lists the main controls. Some are true geochemical controls; others are measurement artifacts; several are both.

Operationally, weather primarily affects representativeness for laboratory dry-mass activity, whereas it directly affects in situ dose-rate measurements. The protocol, therefore, separates dry-mass radionuclide activity from field dose-rate dynamics and requires both to be linked to the same sedimentological state.

### 2.4. Sedimentological and Morphodynamic Descriptors as Explanatory Variables

A beach-sediment radioactivity dataset should not be interpreted only by radionuclide and dose variables. The sediment itself must be described quantitatively because the radiological signal is carried by a physical mixture of grains, minerals, voids and bioclastic components [27,28,74]. At minimum, each station should, therefore, report the sampling zone, depth support, dry bulk density, moisture, carbonate/bioclastic fraction, mud fraction, selected grain-size descriptors and, where feasible, heavy-mineral abundance and magnetic susceptibility. These variables transform the statement “activity is higher in this sample” into a testable sedimentological question: is the enrichment caused by finer particles, dense accessory minerals, reduced carbonate dilution, storm sorting, vertical burial, or anthropogenic sediment management?

The grain-size description uses standard sedimentological descriptors rather than new sedimentological theory. Wentworth classes remain the reporting convention for terms such as fine sand, medium sand and granule gravel, while numerical modeling should use continuous or percentile-based descriptors wherever possible [27,28,75,76,77]. The Krumbein ϕ scale and the Folk–Ward mean size, sorting, skewness and kurtosis formulas are provided in Supplementary Table S8 to keep the main text focused on the RadSed-INT decision logic. In the main protocol, these descriptors are used as explanatory covariates for radionuclide partitioning rather than as independent methodological contributions. When complete grain-size classes are used together in regression, the data should be treated as compositional because the fractions sum to one. A reduced set of physically interpretable descriptors, such as $D_{50}$, $\sigma_{I}$, $F_{<63}$, $f_{HM}$ and $f_{CaCO_{3}}$, is often preferable for screening models; if all classes are modeled simultaneously, log-ratio methods should be considered [29,78]. Table 10 lists the minimum descriptors that should accompany radionuclide data in a defensible implementation.

For predictive interpretation, activity should be modeled as a function of physically meaningful sedimentary covariates rather than as a simple bivariate correlation with one grain-size class. A practical mixed model is

(50) $$\log C_{j}=\beta_{0j}+\beta_{1j}M_{z}+\beta_{2j}\sigma_{I}+\beta_{3j}F_{<63}+\beta_{4j}f_{HM}+\beta_{5j}f_{CaCO_{3}}+\beta_{6j}\kappa +u_{t,j}+u_{q,j}+\epsilon_{j},$$

where $C_{j}$ is the activity concentration of radionuclide *j*, $u_{t,j}$ is a transect-level random effect, $u_{q,j}$ is a campaign-level random effect and $\epsilon_{j}$ is residual variation. Equation (50) is not proposed as a universal law. It is a transparent starting model that separates textural, mineralogical, dilution and temporal controls. If all grain-size classes are used simultaneously, compositional data methods should be applied because particle-size percentages are constrained to a constant sum [29]. When a reduced model is desired, RadSed-INT recommends using a small set of interpretable variables–for example $M_{z}$, $\sigma_{I}$, $F_{<63}$, $f_{HM}$, $f_{CaCO_{3}}$ and κ–rather than all sieve classes as independent predictors. Regression, trend and mixed-effect models should not be fitted after naive substitution of left-censored observations, such as universal MDA/2 replacement; censored-data methods or a clearly stated quantified subset should be used instead.

The sedimentological part of the protocol also requires a morphodynamic context. Repeated surveys should document whether points are fixed by geographic coordinates, by morphological position, or by both. A fixed global positioning system (GPS) point may move from dry backshore to active beachface after erosion, whereas a morphodynamic point labeled “berm crest” may migrate landward or seaward through time. For this reason, the sampling record should include beach profile position, recent wave climate, storm occurrence, grooming, nourishment and visible sedimentary structures. Beach morphodynamics and grain-size variability are not background noise; they are part of the mechanism by which radionuclide-bearing layers are concentrated, buried, exhumed or diluted [51,52,53,74,79,80,81,82].

### 2.5. The RadSed-INT Protocol

#### 2.5.1. Overview

**Figure 4:**
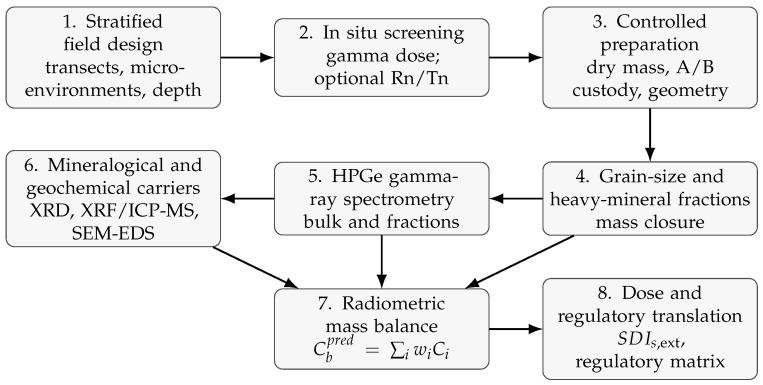
Consolidated operational workflow of RadSed-INT. The figure links the eight-stage field–laboratory sequence with the tiered escalation logic: field screening and sampling define the sediment population, HPGe and grain-size analysis support bulk–fraction closure and the dose/regulatory step activates Tier 4, Tier 5 or S4 modules only when their triggers are met.

RadSed-INT is organized as an eight-stage core workflow (Figure 4), with optional specialist modules for vertical radiostratigraphy and aeolian resuspension when triggered by the decision rules. The principle is simple: every radiological statement must be traceable backward to a physical sediment population and forward to an exposure scenario. The protocol, therefore, avoids uncontrolled compositing, forces mass accounting during fractionation, measures bulk and fractions in compatible geometries, and reports dose in a way that can be translated into national regulatory systems. Mineralogical abbreviations used in Figure 4 are defined here: XRD denotes X-ray diffraction, XRF denotes X-ray fluorescence, ICP-MS denotes inductively coupled plasma mass spectrometry, and SEM-EDS denotes scanning electron microscopy with energy-dispersive X-ray spectroscopy.

#### 2.5.2. Stage 1: Sampling Design

Sampling must begin with a sedimentological model of the site. A beach should be divided into units that are meaningful for sediment supply and human exposure: river-mouth influence, nourished sector, eroding sector, accreting spit, black-sand lens, carbonate beach, siliciclastic beach, tourist zone, maintenance zone and reference area [74,83]. Within each unit, samples should be collected along cross-shore transects that include, when present, the backshore, berm, dry recreational beach, swash zone and intertidal zone. A practical minimum is three true field replicates per micro-environment, not merely three laboratory subsamples of a single composite.

The default surface bulk interval is 0 cm to 5 cm, because this support is comparable with many environmental sediment studies and normally provides sufficient dry mass for HPGe counting. However, RadSed-INT treats this interval as a practical surface bulk, not as a universal definition of the radiologically active surface. Where recent deposition, visible black-sand films, artificial fallout, contact exposure or dose-rate anomalies are relevant, an additional 0 cm to 2 cm veneer sample should be collected. The routine subsurface check is 5 cm to 20 cm; it identifies whether enrichment is limited to the surface or persists below it. Where vertical stratification is suspected, the Tier 4 mini-core interval scheme should replace the broad subsurface check so that a 5–8 cm buried layer is not diluted into a 5–20 cm composite. GPS position, elevation or tidal context, moisture state, visible heavy-mineral laminae, recent nourishment or grooming, and meteorological conditions should be recorded. If a composite is used, it must be a declared composite with a specified number of increments, mass per increment and spatial support. These requirements are consistent with marine-sediment sampling guidance, which emphasizes sampling strategy, sampling devices, field observations, handling, packaging and storage as integral parts of the environmental result [60,83].

#### 2.5.3. Confirmatory A/B Pairs, Custody and Minimum Mass

For exploratory mapping, a single well-documented primary sample at each station may be sufficient. For any anomaly, regulatory decision, high-public-use sector, suspected hot spot, or sample likely to be used in public communication, RadSed-INT requires paired primary/confirmatory A/B sampling analogous to forensic duplicate retention. The A sample is analyzed first. The B sample is sealed, labeled, archived under documented custody and analyzed only for confirmation, inter-laboratory comparison, or dispute resolution. This requirement is intended to separate field heterogeneity, splitting and preparation uncertainty, and counting uncertainty, which, otherwise, may be conflated in a single bulk result.

Two different duplicates are, therefore, distinguished. A co-located field duplicate is an independently collected sample from the same station, depth interval and sedimentary support, placed in a separate container. It tests field-scale heterogeneity. A laboratory split duplicate is obtained after drying, disaggregation and homogenization of the same field sample. It tests splitting, preparation and counting reproducibility. The two are not interchangeable. This distinction is consistent with the Multi-Agency Radiological Laboratory Analytical Protocols Manual (MARLAP), which requires that sampling, preservation, tracking and laboratory preparation be defined before analysis, and with particulate-sampling principles showing that a subsample is unbiased only when every particle has a known opportunity to enter the aliquot [84,85,86]. For Tier 1, at least 10% of stations should have field duplicates and at least 10% of laboratory preparations should be split duplicates. For Tier 2 and above, all triggered anomaly stations should have a field A/B pair; Tier 3 regulatory or management samples should also retain a sealed archive mass sufficient for independent reanalysis. Independent laboratory verification is required before final interpretation when a result affects a management or regulatory classification and at least one of the following conditions occurs: the A/B statistic fails the data-quality objective, gamma-line consistency is poor, the bulk–fraction closure test fails, field dose-rate measurements and HPGe-derived expectations disagree strongly, artificial radionuclides or localized high-activity particles are suspected, or the result lies close to the selected decision threshold or dose tier. Discrete high-activity particles may invalidate homogeneous-bulk assumptions for local dose and representativeness, even when the bulk activity concentration appears moderate. In less consequential exploratory cases, the same checks remain recommended good practice.

The A/B design supports forensic retention, dispute resolution and interlaboratory verification; it does not replace routine laboratory quality-control replicates, split duplicates, blanks, or reference-material checks required by the laboratory quality system.

The retained B sample is, therefore, not a single object but a declared retention state. RadSed-INT distinguishes five possible retained materials: the raw field retained sample $B_{F}$, the dried and homogenized analytical split $B_{D}$, the sealed counting-geometry archive $B_{G}$, the wet witness aliquot $B_{W}$, and, where Tier 4 stratigraphy is triggered, the vertical segment archive $B_{V}$. For routine total gamma-emitting radionuclides, the primary confirmatory reanalysis material is $B_{D}$, because the measurand is normally expressed on a dry-mass basis and because a dried, homogenized split minimizes moisture drift, biological alteration and short-range grain segregation. The raw field retained sample $B_{F}$ is retained when the original sediment fabric, visible lamination, shell content, or dispute over field representativeness may matter. A wet aliquot $B_{W}$ is not the default long-term archive; it is collected only when field moisture, pore water, radon/thoron exhalation, salt crusts, sediment fabric, or emergency wet-counting are part of the objective. The vertical segment archive $B_{V}$ is retained when a buried lamina, mini-core interval, or stratigraphic support is part of the decision. This separation prevents a common ambiguity: a reanalysis of a dried homogenate tests the laboratory result, whereas a reanalysis of a raw wet jar or a vertical slice may test a different sediment microvolume.

Duplicate consistency is evaluated with both a percentage relative difference and an uncertainty-normalized contrast:

(51) $$RPD_{AB,j}\left(\%\right)=100\;\frac{2\left|C_{A,j}-C_{B,j}\right|}{C_{A,j}+C_{B,j}},$$

(52) $$Z_{AB,j}=\frac{C_{A,j}-C_{B,j}}{\sqrt{u^{2}\left(C_{A,j}\right)+u^{2}\left(C_{B,j}\right)-2\;\mathrm{cov}\left(C_{A,j},C_{B,j}\right)}}.$$

The covariance term should be retained when A and B share calibration, geometry, splitting, or counting components; the diagonal form is used only when shared components are unavailable or negligible. Values $|Z_{AB,j}|\leq 2$ indicate consistency at approximately the 95% level under normal uncertainty assumptions. If $|Z_{AB,j}|>2$ and $RPD_{AB,j}\left(\%\right)$ exceeds the project data-quality objective, the result is not automatically rejected; instead, the cause must be assigned to real heterogeneity, inadequate homogenization, geometry mismatch, loss of fines, insufficient counting precision or an erroneous anomaly classification. In high-stakes cases, the B sample should be counted in a second calibrated geometry or by an independent laboratory.

##### Preservation of the B Sample: Dry, Raw, Wet, Sealed and Vertical States

The default preservation route for a confirmatory B sample is dry storage after controlled preparation. The parent field material is first documented, weighed wet, dried or freeze-dried according to the stated objective, disaggregated without losing fines, homogenized, and split by a method that reduces segregation, preferably riffle splitting, rotary splitting, or incremental splitting rather than simple coning and quartering. The analytical A portion and the retained $B_{D}$ portion must have the same grain-size support, the same removal policy for shells or foreign bodies, and the same dry-mass basis. MARLAP explicitly treats drying, constant weight, grinding, sieving, mixing and subsampling as operations that affect the representativeness of solid soil and sediment aliquots; the Japanese emergency gamma-spectrometry guide similarly specifies drying sea sediment to constant mass at 105 °C for dry-rate determination and recommends low-humidity storage of dried samples [85,87]. In RadSed-INT, the dried $B_{D}$ archive is stored in a clean polyethylene, polypropylene, glass, or polytetrafluoroethylene (PTFE)-lined container, double-bagged if necessary, protected from dust and salt contamination, kept in a low-humidity cabinet or desiccator, and logged in a secure sample register. No chemical preservative is added to sediment intended for gamma-ray spectrometry.

Wet preservation is treated as an exception, not as the default retained B sample. A wet $B_{W}$ aliquot may be necessary when the original water content, radon/thoron emanation, pore-water chemistry, biological state, or intact sedimentary fabric is itself part of the measurement question. It should be placed in a completely filled or otherwise well-defined gas-tight container, kept dark and cool during transport, and processed as soon as practicable. For longer storage of marine sediments before gamma analysis, freezing at approximately −5 °C to −20 °C and subsequent freeze-drying is used in established marine monitoring procedures, whereas urgent monitoring may count wet sediment directly in a suitable geometry [88]. These alternatives must not be mixed silently. If a wet sample is later dried, the result belongs to the dry-mass gamma protocol; if it is used for radon/thoron exhalation, drying or freezing may have changed permeability, moisture-controlled emanation and gas transport. RAD7 continuous radon/thoron monitor screening is particularly sensitive to humidity in the measurement chamber, so relative humidity, desiccant condition, purge procedure and measurement geometry must be recorded whenever wet aliquots are used for exhalation work [89].

For ^226^Ra determined through ^222^Rn progeny, a third state is required: the sealed counting geometry $B_{G}$. Once a prepared dry aliquot is transferred into a Marinelli beaker, cylindrical vial or other calibrated geometry, the container is sealed in a radon-retentive manner, labeled with the sealing date and retained without opening until ingrowth is complete or explicitly corrected. If the container is opened, repacked, or transferred, the ingrowth clock restarts. The first-order ingrowth factor for radon supported by radium, i.e., the fraction of equilibrium activity reached at sealing time $t_{s}$, may be written as

(53) $$F_{Rn}\left(t_{s}\right)=1-\exp\left(-\lambda_{Rn}t_{s}\right),$$

where $t_{s}$ is the time elapsed since sealing and $\lambda_{Rn}=\ln2/T_{1/2,Rn}$ with $T_{1/2,Rn}=3.823\;d$. Thus $F_{Rn}$ is approximately 0.98 after 21 d, 0.996 after 30 d, and 0.999 after 40 d. A conservative RadSed-INT procedure, therefore, retains sealed Ra-counting archives for at least 30 d to 40 d before confirmatory counting unless a validated non-equilibrium correction is applied. Studies and technical procedures for radium-bearing residues and sediment gamma-ray spectrometry commonly use leakproof containers and storage periods of about 30 days or longer to establish equilibrium between ^226^Ra and short-lived radon progeny [88,90].

The custody requirement is as important as the physical preservation. Each A/B pair is assigned a permanent identifier at collection, with sample code, station, transect, depth interval, date, collector, wet mass, photographs, container type, preservation state and requested analyses. The B container is closed with a tamper-evident seal placed across the lid or outer bag, not merely labeled on the side, and every transfer is recorded with date, time, relinquishing party and receiving party. At laboratory receipt, seals, leakage, sample identity, matrix, mass and preservation state are inspected before analysis; nonconformances such as broken seals, leaking containers, missing custody forms, or altered physical appearance are recorded before the sample is accepted. This follows the MARLAP approach in which chain-of-custody maintains physical possession or control from collection to disposal, and custody seals are specifically used to reveal unauthorized tampering [91]. For publication-quality baseline studies, $B_{D}$ should be retained at least until peer review and data release are complete. For regulatory, disputed, or management-triggering samples, RadSed-INT recommends retention for a project-defined period, normally not less than five years and preferably ten years where national law or contractual obligations do not prescribe a longer period.

Table 11 summarizes the decision. Operationally, $B_{D}$ is retained for dry-mass gamma confirmatory reanalysis, $B_{F}$ for field representativeness, $B_{W}$ only for objectives that require the wet state, $B_{G}$ for radium by progeny in a sealed geometry, and $B_{V}$ for intact or interval-specific vertical radiostratigraphy.

The required field mass is determined by the counting geometry, the fractionation plan and the need to retain an archive. A useful planning equation is

(54) $$m_{\mathrm{field}}\geq \frac{m_{\mathrm{HPGe}}+m_{\mathrm{frac}}+m_{\mathrm{geo}}+m_{\mathrm{arch}}}{\left(1-f_{w}\right)\left(1-f_{L}\right)},$$

where $m_{\mathrm{field}}$ is the wet mass collected, $f_{w}$ is expected water mass fraction, $f_{L}$ is expected processing loss, $m_{\mathrm{HPGe}}$ is dry mass required to fill the selected counting geometry, $m_{\mathrm{frac}}$ is dry mass required for grain-size and heavy-mineral work, $m_{\mathrm{geo}}$ is dry mass for geochemical/mineralogical analysis, and $m_{\mathrm{arch}}$ is the retained B or archive mass. Table 12 gives defensible starting quantities. They must be adjusted to local grain size, moisture and detector geometry.

#### 2.5.4. Stage 2: In Situ Gamma Survey and Radon/Thoron Screening

Laboratory HPGe data should be paired with in situ gamma-dose-rate measurements. Measurements at 1 m above the sediment support comparison with dose-rate models; contact measurements help identify thin layers and localized heavy-mineral patches. A walking gamma survey or grid survey is recommended where beach heterogeneity is strong. Instruments must be calibrated, energy compensated where applicable, and controlled for background and cosmic contributions. Where spectrometric field instruments are available, portable NaI(Tl), LaBr_3_(Ce) or HPGe systems may be used to separate contributions from the uranium series, thorium series, ^40^K and artificial gamma emitters. Field spectrometry has the advantage of averaging a larger sediment volume than a grab sample, whereas laboratory HPGe has the advantage of better geometry control and nuclide-specific accuracy; the two approaches should, therefore, be treated as complementary rather than competing methods [58,92,93]. Several beach studies have shown that natural radioactivity enrichment can be highly localized and linked to sediment sorting rather than to a uniform regional background [11,30,94].

Radon/thoron screening is an optional module and does not replace gamma-ray spectrometry. Commercial continuous radon/thoron monitors such as RAD7 measure radon and thoron through alpha emissions of their short-lived progeny and can be used with soil-gas probes, accumulation chambers, surface emission chambers or water accessories depending on the pathway being investigated [89,95]. In beach sediment, ^222^Rn belongs to the uranium decay-chain segment that includes ^226^Ra, whereas ^220^Rn belongs to the thorium-chain segment, but gas exhalation is controlled by emanation coefficient, grain size, moisture, permeability, pore-water saturation, barometric pressure, wind, tidal water table and chamber sealing. Consequently, RAD7-type measurements should be interpreted as pathway indicators for radon/thoron availability and exhalation, not as direct measurements of ^226^Ra, ^232^Th or ^40^K activity concentration.

For ^222^Rn, if an accumulation chamber of volume $V_{c}$ is sealed over a sediment area $S_{c}$ and the measured concentration is $C_{Rn}\left(t\right)$, a first-order flux model is

(55) $$C_{Rn}\left(t\right)=C_{Rn,0}\exp\left(-\Lambda t\right)+\frac{J_{Rn}S_{c}}{V_{c}\Lambda }\left[1-\exp(-\Lambda t)\right],$$

where $J_{Rn}$ is the surface exhalation flux and Λ is the effective loss constant, including radioactive decay, leakage, back diffusion and instrument-loop losses. Solving for $J_{Rn}$ gives

(56) $$J_{Rn}=\frac{V_{c}\Lambda \left[C_{Rn}\left(t\right)-C_{Rn,0}\exp\left(-\Lambda t\right)\right]}{S_{c}\left[1-\exp(-\Lambda t)\right]}.$$

These expressions follow from the accumulation-chamber mass balance $dC_{Rn}/dt=J_{Rn}S_{c}/V_{c}-\Lambda C_{Rn}$ with initial condition $C_{Rn}\left(0\right)=C_{Rn,0}$. Equations (55) and (56) are not proposed as a universal flux standard; they define the minimum physical variables that must be reported if radon data are used to support the beach-sediment interpretation. Thoron (^220^Rn) requires additional caution because its half-life is only about 55.6 s; chamber geometry, dead volume, tubing delay, surface roughness and the first few centimeters of sediment can dominate the signal. Thoron measurements should, therefore, be treated as specialist pathway indicators and interpreted with a thoron-specific geometry and calibration rather than by direct transfer of a ^222^Rn accumulation model. In routine RadSed-INT applications, the recommended use is pragmatic: perform RAD7 continuous radon/thoron monitor screening at stations with high gamma response, visible heavy-mineral layers, suspected U/Ra or Th-chain enrichment, or exposure scenarios in which inhalation or exhalation may matter; then use HPGe and, where needed, alpha or radiochemical methods for confirmation.

#### 2.5.5. Stage 3: Preparation and Mass Accounting

All laboratory handling must be designed to preserve mass and traceability [83,85]. The sample is weighed wet, dried to constant mass under a documented regime, weighed dry, gently disaggregated, homogenized and split. Drying at 40 °C to 60 °C may be appropriate for mineralogical preservation or organic-rich sediment; drying at 105 °C is common for dry-mass determination and many radioactivity protocols. The chosen temperature must be justified and used consistently. Shell fragments and bioclasts should not be removed unless the scientific question is explicitly restricted to the siliciclastic fraction; if they are removed, their mass must be reported and the resulting measurand must not be called whole-beach sediment.

For ^226^Ra determination by radon progeny, the counted sample must be packed in an airtight container, sealed, and stored for a documented ingrowth period. Marinelli beakers, cylindrical containers, or small vials may be used, but each geometry requires its own efficiency calibration or validated transfer correction. Fill height, density and dry mass are not ancillary details; they are part of the measurand.

#### 2.5.6. Stage 4: Grain-Size Analysis and Heavy-Mineral Fractionation

The minimum particle-size classes are <63μm, 63–125 μm, 125–250 μm, 250–500 μm, 500–1000 μm and 1000–2000 μm, with an optional >2 mm fraction if coarse shell or gravel is relevant. The 63μm boundary is retained because it separates sand from silt-clay in widely used sedimentological classifications and because fine fractions are often treated separately in marine monitoring protocols [27,28,96,97]. The <63μm sediment fraction is a sedimentological fine fraction; it must not be used as a surrogate for PM_10_, inhalable, thoracic or respirable aerosol unless a validated aerosolization, dustiness or size-selection method links the sediment fraction to the airborne particle-size convention. Wet sieving may be necessary for fine material, but all wash water must be recovered, or the mass balance becomes invalid. The protocol requires a mass recovery criterion, for example

(57) $$R_{m}=\frac{\sum_{i}m_{i}}{m_{bulk}}.$$

Values of $R_{m}$ outside 0.98–1.02 require documented investigation. Depending on the cause, the fractionation should be repeated, the uncertainty budget should be expanded, or the sample should be excluded from the bulk–fraction closure claim while still being reported as a bulk result.

For selected samples, the protocol adds density and magnetic separation. The heavy-mineral fraction, commonly separated around ρ>2.8–2.9 g cm^−3^, should be measured radiometrically and characterized by reflected/transmitted light microscopy, X-ray diffraction (XRD), scanning electron microscopy with energy-dispersive X-ray spectroscopy (SEM-EDS), or automated mineralogy. Magnetic and non-magnetic fractions are useful where Fe-Ti oxides, zircon, monazite, allanite and titanite coexist. The aim is not only mineral identification; these observations also parameterize Equation (19). Density and magnetic concentrates are diagnostic subfractions of a parent grain-size class; they are not added to the grain-size closure unless the residual and concentrate masses are both retained in a full hierarchical mass balance.

#### 2.5.7. Stage 5: HPGe Measurement of Bulk and Fractions

The core analytical technique is low-background high-purity germanium gamma-ray spectrometry [23,24,26]. Table 13 lists the minimum radionuclides and gamma lines. The exact line selection depends on detector resolution, background, interferences and sample activity. The measurement time must be selected to achieve detection limits compatible with the regulatory and geochemical questions. For natural radionuclides in ordinary beach sediment, 24–72 h may be required depending on detector efficiency and sample mass. High-activity or high-count-rate samples should not be managed by simply shortening the counting time: dead time, pulse pile-up and related count-rate effects require an appropriate counting geometry, reduced sample mass, increased source–detector distance, matrix dilution or another validated approach that lowers the count rate at the detector.

Quality control must include laboratory blanks, duplicate preparations, duplicate counts, certified reference materials or matrix-matched in-house reference materials, energy and efficiency checks, control charts, and explicit reporting of the decision threshold $y^{*}$, detection limit $y^{\#}$ and any laboratory MDA or reporting limit [26,41,42]. Results below these limits should be reported as left-censored observations, not silently replaced by MDA/2; any descriptive substitution must be declared and must not drive dose decisions, correlations, trend analyses or regulatory interpretation [44]. Because fraction masses may be small, counting geometry may differ between bulk and fraction measurements. In that case, each fraction geometry must be calibrated or modeled; otherwise the mass-balance test in Equation (15) cannot be interpreted.

#### 2.5.8. Stage 6: Mineralogical and Geochemical Carrier Identification

The radiometric data should be coupled to mineralogical and geochemical data, as illustrated by integrated beach-sediment studies combining gamma-ray spectrometry and mineralogical/geochemical characterization [4,35]. At minimum, XRD identifies major crystalline phases; X-ray fluorescence (XRF) or inductively coupled plasma mass spectrometry (ICP-MS) measures U, Th, K, Zr, Ti, Fe, Mn, rare earth elements (REE), Y and Hf and SEM-EDS or microprobe analysis identifies accessory carriers in enriched fractions. The variables Zr, Hf and heavy REE can track zircon; light REE and Th can track monazite; Ti and Fe can track ilmenite, rutile and magnetite; K can track feldspar and micas. The combination of ^232^Th/^238^U, Th/K, Zr/Th, Ti/Zr and REE patterns helps separate provenance from hydraulic sorting.

Data analysis should respect compositional constraints. Grain-size fractions and mineral percentages sum to unity, so ordinary correlations can be misleading. Centered log-ratio or isometric log-ratio transformations are recommended where multivariate models are used [29]. Geochemical background and anomaly thresholds should not be defined by the arbitrary rule mean plus two standard deviations without testing distributional assumptions; robust and exploratory methods are preferable [98].

#### 2.5.9. Stage 7: Radiometric Mass Closure

For each radionuclide *j*, the protocol requires the closure statistic in Equation (15). The closure result is classified as follows: (i) closed, if $|Z_{j}|\leq 2$ and mass recovery is acceptable; (ii) conditionally closed, if $2<|Z_{j}|\leq 3$ but a documented source of extra uncertainty exists; or (iii) failed, if $|Z_{j}|>3$ or if mass recovery is outside the accepted range. Failed closure does not necessarily invalidate the entire study, but it prevents claims about grain-size partitioning until the cause is resolved. Possible responses are re-fractionation, re-homogenization, recounting in a matched geometry, explicit uncertainty inflation, or exclusion of that sample from closure-based interpretation. Closure is assessed for the primary partition used in Equation (14); nested density or magnetic concentrates remain diagnostic unless their parent fraction, recovery and residual mass are included in a full hierarchical mass balance.

The closure plot is a required figure in a full case-study paper. It should show measured bulk activity on the *y* axis and fraction-reconstructed activity on the *x* axis, with a 1:1 line and uncertainty bars. Separate panels for ^226^Ra, ^232^Th, ^40^K, ^210^Pb and ^137^Cs are recommended. This figure is more informative than another table of hazard indices because it shows whether the protocol has controlled the physical mixture.

#### 2.5.10. Stage 8: Dose and Regulatory Translation

The final stage transforms radiogeochemical measurements into a dose statement and then into a jurisdiction-specific interpretation. The recommended order is as follows: (1) compute ${\dot{D}}_{\gamma }$ from Equation (22); (2) compare ${\dot{D}}_{\gamma }$ with in situ measurements; (3) define occupancy scenarios; (4) decide whether the comparison uses total site-related dose, incremental dose above local or regional background, or incremental dose caused by handling/reuse; (5) select and declare $E_{ref,s}$ according to Table 4; (6) compute $E_{s,site}^{\mathrm{ext}}$ or $\Delta E_{s}^{\mathrm{ext}}$ and the corresponding $SDI_{s,\mathrm{ext}}$; compute $SDI_{s,\mathrm{tot}}$ only if triggered internal pathways are quantified; (7) select the regulatory module according to the sediment scenario and (8) report conventional indices only as supplementary comparators. This order prevents a common interpretive weakness: using a product index or a NORM clearance value as if it were a direct beach standard. The S4 trigger is operational: if ^137^Cs, ^134^Cs, ^60^Co, ^241^Am, or other artificial radionuclides are detected above decision criteria or site-specific background, the case is transferred to S4 irrespective of the natural-radionuclide dose estimate. HPGe may identify the trigger, but it may not resolve the decision question. ^90^Sr, Pu isotopes, ^210^Po or discrete high-activity particles may require radiochemical separation, alpha spectrometry, beta counting, autoradiography, particle screening or contact/skin-dose assessment before any final classification. Supplementary Table S6 organizes the S4 workflow into fallout-gamma, discrete-particle and non-gamma/radiochemical branches.

### 2.6. Use of AI-Assisted Tools in Manuscript Preparation

AI-assisted tools were used during manuscript preparation for language refinement, LaTeX formatting support and preparation of a preliminary draft of the graphical abstract. They were not used to generate primary data, perform calculations, derive equations, select regulatory criteria, conduct independent scientific interpretation, or determine the conclusions of the study. All scientific content, equations, references, regulatory statements and final graphical material were reviewed, edited and approved by the authors, who take full responsibility for the final manuscript.

## 3. Protocol Outputs and Decision Products

Because the present paper is methodological and does not report new primary field data, this section presents protocol outputs and decision products rather than empirical results. The output of RadSed-INT is not a single concentration table. It is a structured set of decision products that connect field observations, laboratory measurements, sedimentological evidence and dose interpretation. In a complete implementation, the minimum outputs are an in situ gamma-dose-rate map or transect profile, a table of A/B pairs and preservation states, HPGe activity concentrations with uncertainty and decision thresholds, and sedimentological descriptors with grain-size statistics. The same package should include a bulk–fraction radiometric closure plot, vertical radiostratigraphic profiles where triggered, aeolian-resuspension and inhalation screening outputs where the pathway is activated, and scenario-specific dose and regulatory screening statements. The Supplementary Material completes this reporting package by preserving the extended notation, extended regulatory matrix and full confounder checklist that are too detailed for the main text but necessary for transparent implementation. These outputs are designed to be auditable. A reviewer can see what activity was measured, why that sample was collected, whether it represents the intended sedimentary population and whether the result has been confirmed.

### 3.1. Secondary-Data Worked Example: Scenario-Dependent Interpretation

The ID5 beach-sediment dataset of Caridi et al. provides a compact demonstration of the protocol logic without introducing new primary data [4]. The reported activities, $C_{Ra}=60.2\;\mathrm{Bq}\;{\mathrm{kg}}^{-1}$, $C_{Th}=524\;\mathrm{Bq}\;{\mathrm{kg}}^{-1}$ and $C_{K}=148\;\mathrm{Bq}\;{\mathrm{kg}}^{-1}$, give I≈2.87 when inserted into the European building-material activity concentration index. This output is relevant only if the sediment is treated as a material or aggregate. For sediment left in situ, the same published dose rate of about $351\;\mathrm{nGy}\;h^{-1}$ gives approximately $0.106\;\mathrm{mSv}\;a^{-1}$ for $432\;h\;a^{-1}$ of beach occupancy, and about $0.49\;\mathrm{mSv}\;a^{-1}$ for a $2000\;h\;a^{-1}$ high-occupancy maintenance scenario. The measured activities do not change; the decision product changes because material status, occupancy and regulatory domain change. If $E_{ref}=0.3\;\mathrm{mSv}\;a^{-1}$ is used only as an internal investigation level, the same dose rate gives $SDI_{tour,\mathrm{ext}}\approx 0.35$ for the tourist scenario and $SDI_{work,\mathrm{ext}}\approx 1.64$ for the high-occupancy maintenance scenario. Because the published dataset does not include activity by grain-size class, a RadSed-INT bulk–fraction closure test cannot be performed; this absence is itself instructive, because it shows what additional information a full protocol implementation would add. This worked example is the practical reason for separating S1–S4 before applying thresholds, indices, or dose tiers.

As a purely arithmetic closure template, a future case study with three fractions could report, for one radionuclide, w=(0.20,0.50,0.30) and $C=\left(40,80,200\right)\;\mathrm{Bq}\;{\mathrm{kg}}^{-1}$. The reconstructed bulk value would be $C_{b}^{pred}=0.20\times 40+0.50\times 80+0.30\times 200=108\;\mathrm{Bq}\;{\mathrm{kg}}^{-1}$. If the directly measured bulk value were $C_{b}^{meas}=110\;\mathrm{Bq}\;{\mathrm{kg}}^{-1}$ and the combined standard uncertainty were $u_{c}=9\;\mathrm{Bq}\;{\mathrm{kg}}^{-1}$, the closure contrast would be Z=(110−108)/9=0.22. The numerical values are not field data; they illustrate how the protocol converts a qualitative grain-size statement into a reproducible mass-balance check.

### 3.2. Regulatory Translation as a Protocol Output

#### 3.2.1. Four Scenarios That Must Not Be Conflated

**Figure 5:**
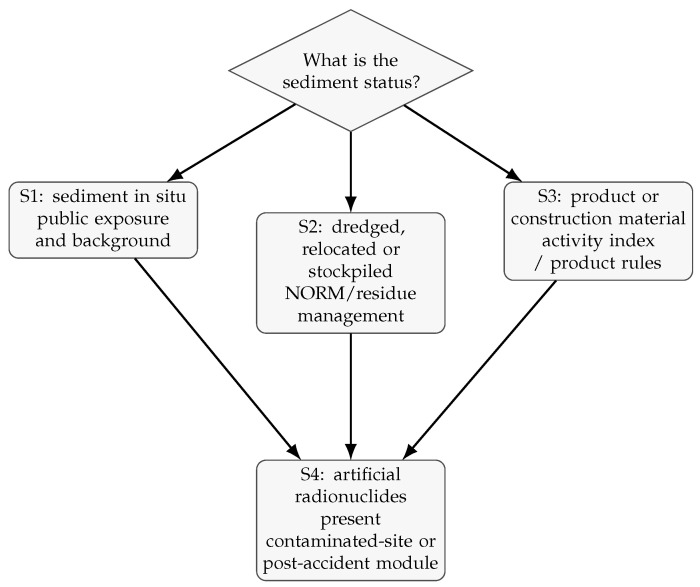
Regulatory decision tree. A beach sediment activity concentration has no unique regulatory meaning until the sediment status and exposure scenario are identified.

The same sediment may pass through different regulatory identities. Four scenarios are defined (Figure 5).

S1 is natural beach sediment left in situ. The relevant question is external exposure of representative individuals and, where appropriate, dust inhalation or radon/thoron exhalation. S2 is sediment that is dredged, relocated, stockpiled, treated, or disposed of; management rules, NORM concepts and dust-generating handling pathways may apply. S3 is sand used as a product, construction material, or industrial raw material; product-specific indices and standards become relevant. S4 is sediment containing artificial radionuclides above decision criteria or site-specific background; contaminated-site, emergency, remediation, or waste frameworks may apply. S4 also covers cases in which non-gamma emitters or localized particles are plausible enough to require methods beyond routine HPGe, including ^90^Sr, Pu isotopes, ^210^Po, autoradiography, particle screening or contact/skin-dose assessment. The scenario matrix in Table 14 summarizes the decision logic used in the main text. The extended multi-jurisdictional matrix is provided as Supplementary Table S2 because national provisions are date-sensitive and cannot be treated as fixed scientific constants. At the time of application, the assessor must verify the current jurisdictional instrument, material status and exposure pathway before using any numerical criterion.

#### 3.2.2. A Default Protocol for Jurisdictions Without Beach-Specific Criteria

In the absence of national beach-sediment radioactivity criteria, the protocol recommends a tiered screening approach (Table 15). The tiers are deliberately dose-based and are used for investigation and prioritization, not as regulatory limits, permitting criteria, public-access restrictions, or public-communication thresholds. They must be read together with the dose-quantity declaration in Table 4: $E_{s}^{\mathrm{ext}}$ is an incremental exposure only if a local background or pre-intervention baseline has been explicitly subtracted; otherwise, it is a site-related external dose for the declared scenario. Activity concentrations of 1 Bq g^−1^ for U/Th-series radionuclides and 10 Bq g^−1^ for ^40^K are used as generic NORM screening triggers derived from international exemption/clearance and graded-approach concepts for solid materials; they are not proposed as beach-specific regulatory limits or safety thresholds for recreational sediment left in situ [8,20,99]. The tiers also require a separate artificial-radionuclide screen. If ^137^Cs, ^134^Cs, ^60^Co, ^241^Am or other artificial radionuclides are detected above decision criteria, the case moves to S4 regardless of natural radionuclide dose.

### 3.3. Validation Strategy for Future Applications

Because this article is methodological and does not report new primary field data, empirical validation is defined as an implementation requirement for future case studies rather than as a result claimed here. A future application should test five measurable properties of the protocol. First, the measured bulk activity should close with the mass-weighted fraction reconstruction within combined uncertainty for the radionuclides that control dose or classification. Second, A/B confirmatory samples should meet project-defined relative percent difference and uncertainty-normalized criteria, or the source of disagreement should be identified. Third, field dose-rate patterns should be explainable from HPGe, depth structure, moisture, density and geometry; unresolved field–laboratory mismatch should trigger Tier 4 or particle-specific investigation. Fourth, vertical mini-cores should detect or rule out buried enriched laminae in sites where surface processes make them plausible. Fifth, Tier 5 should be evaluated only in settings where dust generation is plausible, and the selected particle-size convention, airborne mass estimate and inhalation coefficients must remain coupled. These validation metrics allow a case-study paper to test whether RadSed-INT improves representativeness, metrological traceability and scenario interpretation rather than merely adding analytical detail.

### 3.4. Role of the Supplementary Materials

The Supplementary Materials are part of the RadSed-INT implementation package and are cited from the main text where they support reproducibility. They do not introduce additional scientific claims beyond the main manuscript. Supplementary Table S1 expands the notation used in the equations and reporting templates; Supplementary Table S2 provides the extended, illustrative and date-sensitive regulatory translation matrix; Supplementary Table S3 provides the extended checklist of environmental, geomorphic and methodological confounders; Supplementary Table S4 provides the HPGe correction and quality-assurance checklist; Supplementary Table S5 provides uncertainty-propagation schemes for radiometric closure; Supplementary Table S6 provides an S4 workflow for artificial radionuclides and hot particles and Supplementary Table S7 provides Tier 4/Tier 5 investigation triggers. These materials are implementation aids. In particular, Supplementary Table S2 is not a regulatory instrument and must be checked against current primary legislation and competent-authority guidance before any case-specific application.

### 3.5. Minimum Reporting Requirements

The minimum reporting set is given in Table 16. A compact template for reporting future case-study bulk and fraction data is provided in Appendix B. A manuscript that omits these elements may still be useful as a preliminary survey, but it should not claim to provide a complete radiogeochemical or regulatory assessment. For full protocol documentation, this table should be read together with Supplementary Tables S1–S3, which document extended symbols, regulatory translation details and field/laboratory confounders.

## 4. Discussion

### 4.1. Why Bulk-Only Assessment Is Often Insufficient

Bulk activity is necessary, but it is rarely sufficient. A bulk value can be interpreted only after asking what has been averaged [4,28,30]. If a sample is dried, sieved to <2 mm and packed in a Marinelli beaker, the result represents a laboratory-defined fraction of the beach material. If heavy minerals occur as thin laminae, the measured bulk value may depend on whether the sampler happened to include or exclude that lamina. If the fine fraction is lost during washing, ^137^Cs and ^210^Pb may be underestimated. If density changes between bulk and fractions, efficiency calibration can bias the mass closure. These are not philosophical concerns; they are routine physical mechanisms in beach sediment.

The Camargue example is instructive because enrichment of U and Th was linked to heavy-mineral content and grain-size sorting, with the <200μm fraction carrying a large share of the radionuclide activity [30]. Studies from Brazil, India, Bangladesh, Greece, Egypt and southern Italy show comparable principles in different geological settings: natural radionuclides are not randomly distributed in sand but are associated with sediment provenance, mineral assemblages and hydraulic concentration [2,3,4,9,10,13,31,94]. The contribution of RadSed-INT is to turn that observation into a mass-balance requirement rather than leaving it as a qualitative explanation.

### 4.2. Reframing the Grain-Size–Radioactivity Relation

The conventional phrase “fine particles have higher radioactivity” should be replaced by a more precise statement [27,29,30]: radionuclide activity partitions among grain-size classes according to the mass fractions of radionuclide-bearing mineral and surface-associated carriers. In some environments, clays and Fe-Mn oxides dominate. In other environments, fine to medium sand enriched in zircon and monazite dominates. In carbonate beaches, the radiogenic component may be a minor siliciclastic or heavy-mineral admixture diluted by bioclasts. The sign and strength of the grain-size relation, therefore, depend on mineralogy.

This reframing also has statistical consequences. Because the size fractions sum to unity, correlations between activity and one fraction can be induced by closure even when no physical relationship exists. Compositional data methods should, therefore, be preferred for multivariate modeling [29]. A robust case-study paper should not stop at Pearson correlations between $D_{50}$ and activity concentration. It should model activity as a function of mass-normalized fractions, heavy-mineral abundance, mineralogical ratios and geochemical tracers, and it should validate the model through the closure relation in Equation (14).

### 4.3. Radiological Endpoint and Chemical-Toxicity Boundary

The protocol deliberately separates radiological toxicity from chemical toxicity [6,7,8]. Uranium, thorium, rare earth elements, Fe–Ti oxides and associated metals may have chemical or ecotoxicological relevance in particular settings, but those endpoints require bioaccessibility, solubility, speciation, intake and toxicological reference values that are not provided by HPGe activity concentrations alone. RadSed-INT, therefore, uses mineralogical and geochemical measurements to identify radionuclide carriers, explain partitioning and support dose assessment, not to infer chemical toxicity. Where a beach is also suspected to be chemically contaminated, the radiological protocol should be coupled to a separate chemical-risk framework rather than folded into a single undefined hazard index.

### 4.4. The Role of Conventional Hazard Indices

Conventional indices have two legitimate uses. First, they allow comparison with earlier studies. Second, some indices are embedded in product-specific regulatory frameworks, especially for building materials. They become problematic when treated as universal regulatory criteria. The European building-material activity concentration index in Equation (2) is meaningful for materials incorporated into buildings, because the exposure geometry, occupancy and shielding assumptions are those of buildings [16,17,18]. The exposure geometry of a beach differs from that of a building. A tourist beach, a nourished beach, a mineral-sand stockpile and an aggregate product are four different exposure situations.

A second limitation is statistical rather than regulatory. A long list of radiological indices does not necessarily provide a richer assessment. Many reported quantities—absorbed dose rate, annual effective dose or AEDE, $Ra_{eq}$, $H_{ex}$, $H_{in}$, *I*, AGED and ELCR—are deterministic transformations of the same small vector $\left(C_{Ra},C_{Th},C_{K}\right)$, sometimes with only a scalar occupancy or risk factor added. Mutual correlations among such indices, therefore, often express algebraic coupling rather than independent environmental evidence. Published index suites, including studies on Calabrian sediments [5], are most useful when the indices are treated as scenario-specific descriptors rather than independent lines of proof. RadSed-INT accordingly assigns greater inferential weight to variables that are physically independent of the index formulas: grain-size distribution, heavy-mineral fraction, vertical position, field dose-rate pattern, sample preservation state, mineralogical carriers and A/B confirmation.

The SDI family does not replace regulatory judgment; it makes the comparison explicit by linking the selected dose criterion, representative individual and occupancy time. A high activity concentration can produce a low annual dose for occasional visitors, whereas a moderate dose rate can become relevant for high-occupancy maintenance scenarios. Internal pathways are treated in the same way: they are added only when inhalation, ingestion, or radon/thoron conditions are measured or conservatively bounded.

### 4.5. Aeolian Resuspension as a Specialist Pathway, Not a Universal Requirement

The aeolian module is used selectively. In RadSed-INT, inhalation is activated only when sedimentology, exposure and meteorology support it. The relevant question is not whether the bulk sediment contains radionuclides, but whether an inhalable, thoracic or respirable fraction is present, enriched, dry and mobile. A first-pass inhalation screening estimate requires fine-fraction activity measurements, dust or PM information, and explicit exposure variables before estimating $E_{s,ann}^{\mathrm{inh}}$ [32,33,63,66,67]. The module is particularly defensible for dry backshore and dune systems, mechanically groomed tourist beaches, stockpiled dredged or nourished sediment, mineral-sand accumulations and post-event surfaces where fine contaminated sediment can be deflated. It is generally unnecessary for saturated swash-zone sand unless that material is later dried, handled, or stored.

### 4.6. Artificial Radionuclides and Post-Accident Confusion

Artificial radionuclides require a separate interpretive path [6,8,20]. ^137^Cs is often measured in environmental gamma spectra and may reflect global fallout, regional fallout, sediment focusing, or accident-derived contamination. Its presence does not invalidate a NORM assessment, but it changes the regulatory and radiological framework if it exceeds decision criteria or local background. Post-accident waste criteria, such as radiocesium thresholds developed for specific emergency contexts, should not be imported into natural-radioactivity assessment of beaches unless the sediment is actually part of that post-accident context. Gamma-ray spectrometry alone may be insufficient for some S4 cases: ^90^Sr, Pu isotopes, ^210^Po or discrete high-activity particles can require radiochemical separation, alpha spectrometry, beta counting, autoradiography or particle screening. Localized high-activity particles or artificial beta/gamma emitters may also require contact or skin-dose assessment, which is not represented by ordinary external gamma indices. Such particles may also invalidate homogeneous-bulk assumptions for local dose and sample representativeness. Accordingly, the protocol treats artificial radionuclides as an independent module rather than as additional terms in a generic natural-radioactivity index.

### 4.7. Limits of the Proposed Protocol

RadSed-INT is a tiered screening-to-confirmation protocol. It is not a replacement for full Monte Carlo dose modeling, detailed aerosol physics or dust-dispersion modeling, radon/thoron exhalation studies, ingestion-specific exposure assessment, chemical-toxicity assessment, alpha spectrometry of U and Th isotopes, radiochemical separation of ^210^Pb or ^210^Po, or risk assessment for contaminated sites. It also cannot solve representativeness by mathematical elegance alone. A poorly designed field campaign cannot be rescued by a rigorous laboratory model. The protocol’s strength is that it makes such weaknesses visible: failed mass closure, inconsistent gamma lines, unreported density, missing fine fractions and unclear regulatory scenarios are no longer hidden behind a table of indices.

## 5. Conclusions

Beach-sediment radioactivity should be assessed as both a radiogeochemical mixture problem and a scenario-specific dose problem. Bulk activity concentrations of ^226^Ra, ^232^Th and ^40^K are necessary, but they do not by themselves explain radionuclide distribution or justify regulatory conclusions. RadSed-INT addresses this gap through three linked requirements: sedimentologically stratified sampling, confirmatory HPGe metrology with A/B pairs and radiometric closure, and scenario-specific dose translation.

The scientific contribution of the protocol is the closure equation $C_{b}^{pred}=\sum_{i}w_{i}C_{i}$, which makes the grain-size–radioactivity relation testable. Its radiological contribution is the SDI family, which links activity concentration, occupancy, pathway selection and a declared dose reference instead of treating a single hazard index as universal. Its regulatory contribution is the separation of beach sediment left in situ, moved sediment, product use and artificial contamination into distinct modules. The framework is not a substitute for primary legislation or case-specific dose assessment, but it provides a defensible structure for deciding when a simple survey is sufficient and when spatial, vertical, grain-size, or pathway-specific escalation is required. The Supplementary Materials provide the extended notation, regulatory translation matrix, confounder checklist and trigger-specific implementation tables that support application while keeping the main text focused on the core protocol.

A future case-study implementation should apply the protocol to contrasting beach systems, for example, carbonate, siliciclastic, heavy-mineral-rich, wind-prone backshore/dune and nourished or dredged beaches. Such a dataset would allow the method to be tested across mineralogical and regulatory settings. Until then, the present protocol can serve as a structured template for studies that are reproducible, physically interpretable and less prone to regulatory overstatement. Any dose statement derived within RadSed-INT should explicitly state whether it is site-related or incremental relative to a defined background baseline, which exposure pathways are included, and which representative individual and occupancy scenario are assumed.

## Figures and Tables

**Table 1:** Common radiological quantities and the domain in which they can be defensibly interpreted. The table does not reject the use of conventional indices; it restricts their interpretive weight to the scenarios for which they are meaningful.

Quantity	Defensible Use	Interpretive Limitation
${\dot{D}}_{\gamma }$	External absorbed gamma dose rate in air estimated from terrestrial radionuclides or measured in situ.	Treating a dose rate as an annual effective dose without specifying occupancy, geometry and conversion assumptions.
Annual effective dose (historically reported as AEDE)/$E_{s}^{\mathrm{ext}}$	Scenario-specific annual external effective dose for a defined representative individual.	Using one occupancy factor, such as a summer tourist scenario, to certify all recreational, occupational, or material-use scenarios.
$Ra_{eq}$	Comparative gamma-hazard descriptor, historically used for mixtures of Ra–Th–K in building-material contexts.	Treating the value as a universal regulatory limit for natural beach sediment left in situ.
$H_{ex}$	External-hazard screening index for Ra–Th–K mixtures, mainly for comparison with earlier studies.	Interpreting $H_{ex}<1$ as proof that no scenario-specific dose, dust, or material-use assessment is needed.
$H_{in}$	Indoor/internal screening convention linked to radon-related assumptions in material use.	Applying it to an open beach without defining radon/thoron emanation, enclosure, occupancy, or inhalation pathway.
*I*	Activity concentration index for gamma radiation from building materials under European screening logic.	Using I>1 or I<1 as a direct regulatory classification of a recreational beach surface.
Annual gonadal equivalent dose (AGED)/excess lifetime cancer risk (ELCR)	Comparative risk-related descriptors when assumptions are explicitly stated.	Treating deterministic index values as independent epidemiological evidence or regulatory endpoints.

**Table 2:** Core notation used in the main protocol. The extended notation table is provided in Supplementary Table S1.

Symbol	Quantity	Use in the Protocol
$C_{j}$	activity concentration	Dry-mass activity concentration of radionuclide or decay-chain segment *j*, in Bq kg^−1^.
$x_{h}$	horizontal position	Horizontal position on the beach surface; reducible to cross-shore coordinate in transect applications with alongshore variability represented by replicated transects or explicit stratification.
$N_{\ell }^{net}$	net peak area	Background-subtracted net peak area for gamma line *ℓ* used in HPGe activity estimation.
$\varepsilon_{\ell }$, $P_{\gamma ,\ell }$	efficiency and emission probability	Full-energy peak efficiency and photon emission probability per decay for gamma line *ℓ*.
c, V	line-estimate vector and covariance	Line-specific activity estimates and their covariance matrix in multi-line averaging.
u(X), $u^{2}\left(X\right)$	standard uncertainty and variance	Standard uncertainty of *X* and its variance, i.e., the squared standard uncertainty used in uncertainty propagation.
$w_{i}$	grain-size mass fraction	Dry mass fraction of particle-size class *i*; $\sum_{i}w_{i}=1$.
$C_{bj}^{meas}$, $C_{bj}^{pred}$	measured and reconstructed bulk activity	Directly measured bulk activity concentration and the mass-weighted reconstruction from fractions.
$Z_{j}$	closure statistic	Uncertainty-normalized difference between measured and reconstructed bulk activity.
$D_{50}$, $M_{z}$, $\sigma_{I}$	grain-size descriptors	Median size, Folk–Ward mean size and sorting used as sedimentological covariates.
${\dot{D}}_{\gamma }$	absorbed dose rate	Gamma absorbed dose rate in air, generally in nGy h^−1^.
$E_{s,site}^{\mathrm{ext}}$, $\Delta E_{s}^{\mathrm{ext}}$	external dose quantities	Site-related and incremental annual external effective dose for scenario *s*.
$E_{s,ann}^{\mathrm{inh}}$, $E_{s}^{\mathrm{ing}}$	internal-dose quantities	Annual committed effective dose from inhalation and annual committed effective dose from incidental ingestion for scenario *s*, in mSv a^−1^ when annualized.
$E_{ref,s}$	comparison dose	Declared reference, investigation or screening value used for a specified SDI form.
$SDI_{s,\mathrm{ext}}$, $SDI_{s,\mathrm{tot}}$	Sediment Dose Index forms	External-only and total-pathway ratios between declared dose quantity and selected comparison value.
$y^{*}$, $y^{\#}$	characteristic limits	Decision threshold and detection limit in the radiometric characteristic-limits framework.
$B_{F}$, $B_{D}$, $B_{G}$, $B_{W}$, $B_{V}$	retained B materials	Field, dried, sealed-geometry, wet witness and vertical-segment retained confirmatory materials.

**Table 3:** Core elements and trigger-based diagnostic modules in RadSed-INT. The distinction is editorially and operationally important: Tier 4 and Tier 5 are not routine requirements for every beach.

Level	Minimum Content	Trigger or Decision Role
Core Tier 1–2	Scenario definition, field gamma reconnaissance, representative surface sampling, HPGe dry-mass activity concentration, characteristic limits and basic annual external dose.	Required for exploratory and baseline surveys. It establishes whether the survey can remain a reconnaissance product or must be confirmed.
Core confirmatory step	Paired primary/confirmatory A/B design, chain-of-custody where needed, duplicate preparation/counting and gamma-line consistency checks.	Activated when the result affects a management, regulatory, public-communication, or interlaboratory-verification decision.
Core grain-size closure	Grain-size distribution, selected fraction activity concentrations, mass recovery and $C_{bj}^{pred}=\sum_{i}w_{i}C_{ij}$ closure against the measured bulk.	Core diagnostic criterion of the protocol; diagnoses loss of fines, poor splitting, density/geometry bias, or mineral segregation.
Tier 4 vertical module	Mini-core intervals such as 0–2, 2–5, 5–8, 8–12 and 12–20 cm.	Triggered by visible layering, contact anomalies, erosion, grooming, nourishment, excavation, field–lab mismatch or suspected buried enrichment.
Tier 5 dust/inhalation module	Aerosolizable fraction, airborne mass or resuspension factor, declared particle-size convention, inhalation dose coefficients.	Triggered only when dry fine sediment, dust-generating handling, wind deflation, stockpiles, or artificial/fine-fraction activity make inhalation plausible.
S4 artificial-radionuclide module	Dedicated interpretation for artificial radionuclides, non-gamma emitters and discrete high-activity particles.	Triggered by anthropogenic radionuclides above decision criteria or site-specific background; may require radiochemistry, alpha/beta counting or particle screening.

**Table 4:** Operational choice of $E_{ref,s}$ and of the decision dose quantity. The table does not prescribe regulatory limits; it forces the assessment to state which dose quantity is being compared.

Scenario	Object Evaluated	Defensible Decision Quantity	How $E_{\mathrm{ref},s}$ Is Selected
S1 sediment left in situ	Public or representative beach users	Scenario external dose, with internal pathways added only if triggered.	Use a declared screening or investigation level for the representative individual; specify whether local background has been subtracted. No universal beach-specific regulatory limit is implied.
S2 moved, dredged or stockpiled sediment	Managed material and possible worker/public pathways	Incremental dose caused by handling, relocation, storage, dust generation or reuse.	Select the reference from the applicable NORM, waste, occupational, or material-management framework; evaluate public and worker scenarios separately.
S3 product or construction material	Material placed on the market or incorporated into construction/industrial use	Product-specific screening quantity, such as the building-material activity concentration index or national product criterion.	Use the product or construction-material framework only after the sediment has entered that material status; do not transfer the result back to S1 without redefining the scenario.
S4 artificial radionuclides	Anthropogenic contamination, post-accident material or site-specific fallout	Radionuclide- and pathway-specific contaminated-site, emergency, waste or remediation criterion.	Select the criterion from the relevant artificial-radionuclide framework; do not combine S4 cases with natural-NORM indices without explicit justification.

**Table 5:** Illustrative dose-rate reference values implied by Equation (26). Values are screening quantities, not regulatory limits.

Scenario	$T_{s}$ (h a^−1^)	${\dot{D}}_{\mathrm{ref}}$ at 0.3 mSv a^−1^	${\dot{D}}_{\mathrm{ref}}$ at 1.0 mSv a^−1^
		(nGy h^−1^)	(nGy h^−1^)
Occasional beach user	300	1429	4762
Frequent beach user	700	612	2041
Coastal resident/high recreational use	1000	429	1429
Beach worker or maintenance worker	2000	214	714

**Table 6:** Operational triggers for escalation from exploratory screening to confirmatory tiers. The thresholds are protocol criteria for scientific defensibility, not universal regulatory limits.

Trigger Class	Example Criterion	Protocol Response
Robust field anomaly	$|Z^{rob}|\geq 3$ for contact dose, 1 m dose, gamma count rate, or mapped spectral window.	Collect paired primary/retained-confirmatory samples; repeat field reading; move from Tier 1 to Tier 2 or Tier 3 depending on dose and spatial extent.
Laboratory anomaly	Activity or $SDI_{s,\mathrm{ext}}$ above local background class, yellow/orange/red screening tier, or inconsistent gamma lines.	Confirm with duplicate preparation/count or B sample; fractionate selected samples; evaluate dose and regulatory scenario.
Field–laboratory mismatch	High contact reading but moderate 0–5 cm HPGe activity, or high HPGe activity but low field dose rate.	Check moisture, geometry, background and detector support; consider thin veneers, hot particles, density/attenuation effects, or unrepresented material; activate Tier 4 if depth structure is plausible.
Visual sedimentary evidence	Black-sand lamina, storm lag, erosional scarp, heavy-mineral strandline, or anthropogenic fill.	Targeted surface and depth sampling; density and magnetic separation; mineralogical confirmation.
Temporal forcing	Storm, overwash, nourishment, grooming, excavation, flood sediment pulse, or rapid shoreline change.	Repeat transects after the event and during recovery; compare fixed and morphological coordinates.
Management decision	Dredging, reuse, disposal, beach nourishment, public communication, worker exposure, or regulatory classification.	Tier 3 minimum; paired primary/retained-confirmatory samples; chain-of-custody; jurisdiction-specific regulatory translation.
Aeolian inhalation trigger	Dry fine sediment, windy backshore/dunes, mechanical grooming, scraping, stockpiles, nourishment works, visible dust plumes, or elevated activity in fine sediment or health-related particle-size fractions validated by an aerosolization or size-selection method.	Activate Tier 5; characterize erodibility, airborne mass $M_{x}$, dust activity $C_{x,j}$ and inhalation dose separately.

**Table 7:** Illustrative independent sample sizes from Equation (33) for estimating a stratum mean. Values assume 95% confidence and no clustering. Real designs must use pilot variance and the design effect in Equation (35).

Target Relative Half-Width	CV=0.30	CV=0.50	CV=0.80
r=0.20	9	25	62
r=0.10	35	97	246

**Table 8:** Recommended statistical designs for beach-sediment radioactivity. Fractionated and vertical samples are subsets selected to span low, median and high activity, cross-shore position, grain-size variability and suspected buried enrichment.

Tier	Objective	Minimum Spatial Design	Temporal and Analytical Requirements
1. Tier 1 screen	Identify radiological range, anomalies and need for escalation.	3–5 horizontal transects; 3 cross-shore zones; surface bulk 0–5 cm; 9–15 bulk samples.	One fair-weather campaign; in situ scan; 3–5 fractionated samples; at least 10% field/lab duplicates.
2. Tier 2 baseline	Estimate baseline, cross-shore structure and first depth contrast.	6–8 transects; 4 zones; 0–5 cm surface interval; selected 5–20 cm check interval; typically 24–32 surface bulk samples plus depth subset.	Two campaigns minimum, preferably low- and high-energy seasons; 8–12 fractionated samples; mixed-model variance components.
3. Tier 3 managed site	Support dose screening, nourishment, dredging, worker/public communication, or hot-spot assessment.	10–15 transects; 4 zones; 0–5 and 5–20 cm intervals; adaptive/grid design where patches are expected; typically 80–120 bulk samples.	Four seasonal/event campaigns in the first year, or justified event-based design; 20–30 fractionated samples; site-specific dose and uncertainty analysis.
4. Tier 4 depth profiles	Determine whether enriched units are surface veneers, buried layers, or mixed deposits.	2–4 priority transects plus all anomaly stations; mini-core increments 0–2, 2–5, 5–8, 8–12 and 12–20 cm; optional 20–40 and 40–60 cm.	Triggered by black-sand layers, contact anomalies, storm erosion, nourishment/grooming or management decisions; repeat after erosion and recovery; HPGe on increments and selected fractions.
5. Tier 5 dust pathway	Evaluate whether dry sediment can generate radionuclide-bearing inhalable, thoracic, or respirable dust.	Triggered stations only: dry backshore/dunes, stockpiles, grooming corridors, windy strandlines or validated health-related particle-size fractions; collect 0–1/0–2 cm material and dust/fine fractions.	Measure or estimate $M_{x}$, wind threshold and $C_{x,j}$; calculate $E_{s,ann}^{\mathrm{inh}}$ separately from external-dose screening.

**Table 9:** Main confounder groups to record during RadSed-INT field and laboratory work. The extended checklist is provided as Supplementary Table S3.

Confounder Group	Minimum Reporting Response
Meteorology and moisture	Record rainfall history, sediment moisture, drying state and whether field dose-rate readings were made after rain, washout, or surface wetting.
Wave, tide and storm forcing	Record tidal stage, swash/beachface position, storm erosion, overwash, accretion and whether the sampled unit is morphologically equivalent across campaigns.
Aeolian and dust conditions	Record dry backshore or dune exposure, visible dust, wind, grooming, scraping, stockpiles and any trigger for Tier 5 dust/inhalation assessment.
Sedimentology and stratigraphy	Record grain-size class, shell/carbonate dilution, heavy-mineral laminae, buried layers, anthropogenic fill and the depth support of each sample.
Human management and analytical handling	Record nourishment, dredging, grooming, excavation, sample washing or fine loss, geometry/density changes and deviations from the declared HPGe preparation.

**Table 10:** Minimum sedimentological descriptors recommended for interpreting radionuclide activity in beach sediment. The list separates directly measured variables from derived statistical descriptors and proxy variables.

Descriptor	Symbol	Radiogeochemical Interpretation
Median or mean grain size	$D_{50}$, $M_{z}$	Cross-shore sorting; distinguishes fine, medium and coarse sediment supports.
Sorting	$\sigma_{I}$	Hydraulic selectivity; potential concentration of dense accessory minerals or storm lags.
Skewness and kurtosis	$Sk_{I}$, $K_{G}$	Fine or coarse tails, mixed populations, surface veneers and lag deposits.
Mud fraction	$F_{<63}$	Potential carrier for adsorbed Cs, Pb, Ra, or organic/oxide-bound activity.
Aerosolizable or health-related dust fractions	$F_{x}$, $C_{x,j}$	Optional Tier 5 variables for inhalable, thoracic or respirable dust; tests whether airborne fractions are enriched relative to the bulk.
Gravel/shell fraction	$F_{>2\;\mathrm{mm}}$	Dilution by coarse bioclasts or lithoclasts; changes the definition of the measured sediment.
Heavy-mineral fraction	$f_{HM}$	Direct proxy for zircon, monazite, rutile, ilmenite, magnetite and other dense carriers of U–Th–REE or Fe–Ti phases.
Carbonate/bioclastic fraction	$f_{CaCO_{3}}$	Dilution proxy in carbonate-rich beaches; controls density and attenuation.
Magnetic susceptibility	κ	Rapid proxy for magnetite/ilmenite-rich fractions and Fe–Ti sorting.
Dry bulk density and moisture	$\rho_{d}$, $f_{w}$	Required for in situ gamma interpretation, sample geometry, radon/thoron pathways and vertical profiles.
Morphodynamic zone	$z_{cs}$	Backshore, berm, beachface, swash zone and shoreface are not interchangeable sampling populations.

**Table 11:** Recommended B-sample preservation states in RadSed-INT. The preservation state is part of the measurand and must be reported with the activity result.

B State	Main Purpose	Preservation Procedure	Main Limitation
$B_{D}$: dry split	HPGe confirmatory reanalysis and inter-laboratory repeatability	Dry or freeze-dry to stated endpoint; homogenize; split by controlled method; store sealed, dry and low-humidity	No original moisture, pore gas or intact fabric
$B_{F}$: raw field sample	Field heterogeneity, fabric, shell content, visible laminae	Independent co-located raw sample; photograph; seal; cool/dark transport; process under custody	Different sediment microvolume
$B_{W}$: wet witness	Moisture, pore water, radon/thoron exhalation, emergency wet count	Gas-tight container; defined headspace; cool/dark transport; prompt analysis or documented freezing route	Not a default long-term archive; gas transport may change
$B_{G}$: sealed geometry	^226^Ra through ^222^Rn progeny and geometry-preserved recount	Dry aliquot in calibrated geometry; radon-retentive seal; sealing date; 30 d to 40 d ingrowth before count	Opening or repacking resets ingrowth and changes geometry
$B_{V}$: vertical segment	Buried radioactive layers and Tier 4 radiostratigraphy	Depth-oriented segment or core slice; photographic log; sealed; then process by interval	Low mass may require small-vial calibration and longer counts

**Table 12:** Indicative minimum masses per station. Values are planning quantities, not universal requirements; actual masses depend on detector geometry, moisture, grain-size distribution and archive policy.

Analytical Purpose	Recommended Dry Mass	Comment
Routine bulk HPGe, 0–5 cm	0.5–1.5 kg	Enough to fill common cylindrical or Marinelli geometries; the exact mass is geometry-specific.
Bulk HPGe plus ordinary grain-size distribution	1.5–3 kg	Supports sieving, moisture determination, duplicate split and limited archive.
Full fractionated radiometry and heavy-mineral separation	3–5 kg, more for fine-poor sand	Required because the fine and heavy fractions may be small but radiologically important.
A/B anomaly confirmation	Primary A mass plus independent B mass of comparable support	Retain the appropriate B state: $B_{F}$ for field representativeness, $B_{D}$ for dry-mass confirmatory reanalysis, $B_{G}$ when a sealed Ra ingrowth geometry is needed, $B_{W}$ when wet-state pathways are part of the objective, and $B_{V}$ for Tier 4 vertical segments.
Tier 4 mini-core	Enough mass in each depth increment to meet the selected HPGe geometry or validated small-vial calibration	If increments are thin, small-geometry calibrations and longer counts may be preferable to compositing.

**Table 13:** Minimum gamma-ray spectrometry targets for beach-sediment assessment. Line selection must be validated for each detector and matrix.

Reported Quantity	Typical Gamma Lines/Proxies	Critical Comments
^40^K	1460.8 keV	Direct determination; check interference and background stability.
^226^Ra	^214^Pb 295, 352 keV; ^214^Bi 609, 1120, 1764 keV	Requires sealed container and radon-progeny ingrowth. Report as ^226^Ra unless U-series equilibrium is demonstrated.
^228^Ra/thorium series	^228^Ac 338, 911, 969 keV	Represents ^228^Ra through ^228^Ac equilibrium; line interferences and summing must be considered.
^228^Th/thorium series	^212^Pb 239 keV; ^208^Tl 583, 2614 keV	^208^Tl requires branching and coincidence-summing attention.
^210^Pb	46.5 keV	Mandatory self-attenuation correction or matrix-matched calibration; otherwise report as not quantified.
^137^Cs	661.7 keV	Indicator of artificial fallout or contamination; interpretation is separate from NORM.
Other artificial emitters	e.g., ^60^Co, ^134^Cs, ^241^Am if detected	Trigger the artificial-radionuclide module and, where needed, non-gamma analyses such as radiochemistry, alpha spectrometry, beta counting, autoradiography or hot-particle screening.
Non-gamma or particle-specific targets	e.g., ^90^Sr, Pu isotopes, ^210^Po, discrete high-activity particles	Do not rely on routine HPGe alone; use validated radiochemistry, alpha/beta counting, autoradiography, particle screening or contact/skin-dose assessment when the decision question requires it.

**Table 14:** Scenario-centered regulatory translation matrix. This table is illustrative, non-binding, non-exhaustive and date-sensitive. The extended multi-jurisdictional matrix is provided as a Supplementary Table S2 and must be updated against current primary legislation before application.

Scenario	Object Assessed	Protocol Interpretation
S1	Beach sediment left in situ	Interpret through site-related or incremental external dose for a declared representative individual and occupancy. Dust, radon/thoron and ingestion are added only when triggered. Building-material indices are not used as S1 limits.
S2	Dredged, relocated, stockpiled, treated, or disposed of sediment	Treat as managed material. NORM/TENORM, worker/public handling, dust and waste, or reuse frameworks may apply; the relevant dose quantity and jurisdictional instrument must be declared.
S3	Product, construction material, industrial raw material or aggregate	Apply product- or building-material criteria where relevant, including the building-material activity concentration index *I*. Results must not be transferred back to S1 without redefining material status and exposure.
S4	Sediment with artificial radionuclides or discrete high-activity particles	Use contaminated-site, post-accident, remediation, waste or emergency frameworks. Routine HPGe may be insufficient for ^90^Sr, Pu isotopes, ^210^Po or localized particles.

**Table 15:** Default dose-based screening tiers for countries without beach-specific legislation. The tiers are internal protocol-level investigation and prioritization classes, not legal compliance, permitting, access-restriction, public-communication or regulatory-classification criteria; generic NORM triggers are not direct limits for beach sediment left in situ.

Tier	Screening Condition	Recommended Action
Green	$E_{s}^{\mathrm{ext}}<0.1$ mSv a^−1^ and no artificial radionuclide above decision criteria.	Report as low scenario external exposure for the specified scenario; describe as incremental only if a background or pre-intervention baseline has been subtracted; maintain data as local baseline.
Yellow	$0.1\leq E_{s}^{\mathrm{ext}}<0.3$ mSv a^−1^.	Document site conditions; verify in situ dose-rate agreement; consider repeat measurements if the beach is dynamic or heavily used.
Orange	$0.3\leq E_{s}^{\mathrm{ext}}<1.0$ mSv a^−1^ or strong localized hot spots.	Perform detailed mapping, grain-size closure, heavy-mineral identification, representative-individual assessment and stakeholder communication.
Red	$E_{s}^{\mathrm{ext}}\geq 1.0$ mSv a^−1^ for a plausible representative individual, or artificial radionuclides requiring regulatory action.	Conduct site-specific radiological assessment, consult competent authorities, evaluate management options and optimize exposures.
Dust-pathway trigger	Dry, disturbed or stockpiled sediment with elevated activity in fine or respirable fractions, artificial radionuclides, or plausible public/worker dust exposure.	Activate Tier 5; measure or estimate airborne activity for a declared particle-size convention and add $E_{s,ann}^{\mathrm{inh}}$ separately from external gamma dose if annualized.
NORM trigger	U/Th-series activity ≳1 Bq g^−1^ or ^40^K ≳10 Bq g^−1^ in material to be handled, processed, relocated or used.	Do not classify automatically as unsafe. Treat as a trigger for planned-exposure/NORM evaluation and scenario-specific dose assessment.

**Table 16:** Minimum reporting checklist for RadSed-INT studies.

Component	Required Information
Site and sampling	Coordinates; beach unit; cross-shore position; depth interval; replicate structure; date; tide/weather context; visible heavy-mineral layers; anthropogenic disturbance.
Field radiometry	Instrument; calibration; measurement height; integration time; grid/transect design; background treatment; uncertainty; whether readings were exploratory or confirmatory.
Preparation and custody	Wet mass; dry mass; A/B sample-pair identifiers; B state ($B_{F}$, $B_{D}$, $B_{W}$, $B_{G}$, $B_{V}$); chain-of-custody; archive mass; drying temperature/time; dry-mass basis; disaggregation method; sieving method; removed material; storage humidity/temperature; seal integrity; sealed ingrowth period.
Grain-size analysis	Size classes; mass fractions; mass recovery; method for <63μm; replicate precision; particle-size statistics.
HPGe metrology	Detector; shield; geometry; density; fill height; efficiency calibration; reference materials; live counting time; background; gamma lines; corrections; uncertainty; detection limits; A/B duplicate statistics.
Equilibrium	Waiting time; container seal; gamma-line consistency for ^226^Ra and thorium-chain segments; naming convention for reported radionuclides.
Mineralogy/geochemistry	XRD, XRF/ICP-MS, SEM-EDS or equivalent; carrier minerals; U, Th, K, Zr, Ti, Fe, REE and relevant ratios.
Mass closure	$C_{b}^{meas}$; $C_{b}^{pred}$; $Z_{j}$; mass recovery; enrichment factors; radiometric contributions by fraction.
Dust/resuspension and ingestion modules	If Tier 5 is triggered: particle-size convention; field or laboratory dust-generation method; PM or dust mass concentration; air volume where applicable; wind speed; surface moisture; disturbance source; $C_{x,j}$; breathing-rate assumptions; inhalation dose coefficients. If incidental ingestion is triggered: ingestible fraction; $M_{s}^{ing}$; $C_{j,ing}$; ingestion dose coefficients.
Dose and regulation	Dose coefficients; in situ comparison; occupancy scenarios; SDI form used; tier-escalation status; regulatory scenario S1–S4; jurisdiction-specific interpretation; limitations.

## Data Availability

No new primary field data were generated in this study. The secondary numerical example uses values previously published by Caridi et al. [4]. The Supplementary Materials contain implementation tables only: extended notation, an extended regulatory translation matrix, an extended confounder checklist, HPGe correction notes, closure-uncertainty schemes, an S4 workflow and Tier 4/Tier 5 trigger indicators. Future case-study datasets should be deposited in an open repository together with raw or processed gamma-ray spectrometry metadata, grain-size tables, sample-preservation records and dose-scenario assumptions.

## Supplementary Materials

The following supporting information can be downloaded at https://www.mdpi.com/article/10.3390/toxics14070590/s1, and it is part of the RadSed-INT implementation package: Supplementary Table S1, extended notation used in the equations and decision rules; Supplementary Table S2, extended illustrative, non-binding, non-exhaustive and date-sensitive regulatory translation matrix; Supplementary Table S3, extended environmental, geomorphic and methodological confounder checklist; Supplementary Table S4, HPGe correction and QA requirements; Supplementary Table S5, closure-uncertainty propagation schemes; Supplementary Table S6, S4 artificial-radionuclide operation tree; Supplementary Table S7, Tier 4/Tier 5 investigation triggers; Supplementary Table S8, standard sedimentological equations; Supplementary Table S9, minimal worked example for radiometric mass closure; Supplementary File S1, CSV template for mass closure; Supplementary File S2, Python 3.11-compatible script for mass closure and uncertainty propagation. References [100,101,102,103,104,105,106,107] are cited in the Supplementary Materials and are included in the main manuscript reference list for MDPI submission compliance. These supplementary materials support the abbreviated main-text tables and must be updated against current primary legislation and site-specific conditions before application.

## Abbreviations

A/B	Primary analytical sample and retained confirmatory sample
BSS	Basic Safety Standards
DQO	Data Quality Objectives
HPGe	High-purity germanium detector
MARLAP	Multi-Agency Radiological Laboratory Analytical Protocols Manual
MDA	Minimum detectable activity
NORM	Naturally occurring radioactive material
PM	Particulate matter
PTFE	Polytetrafluoroethylene
QA	Quality assurance
REE	Rare earth elements
RAD7	Commercial continuous alpha-spectrometric radon/thoron monitor or equivalent device
SDI	Sediment Dose Index; reported as external, total-pathway or incremental form
TENORM	Technologically enhanced naturally occurring radioactive material
XRD	X-ray diffraction
XRF	X-ray fluorescence
ICP-MS	Inductively coupled plasma mass spectrometry
SEM-EDS	Scanning electron microscopy with energy-dispersive X-ray spectroscopy

## Appendix A. Derivation of Elemental Concentration-to-Activity Conversions

For a radionuclide *r* in a sample of mass $M_{s}=1\;\mathrm{kg}$, with radionuclide mass fraction $x_{r}$, molar mass $M_{r}$ and half-life $T_{1/2,r}$, the number of atoms is

(A1) $$N_{r}=\frac{x_{r}M_{s}}{M_{r}}N_{A}.$$

The physical activity is

(A2) $$A_{r}=\lambda_{r}N_{r}=\frac{\ln2}{T_{1/2,r}}\frac{x_{r}M_{s}}{M_{r}}N_{A}.$$

For $M_{s}=1\;\mathrm{kg}$, the numerical value of $A_{r}$ in Bq is also the activity concentration $C_{r}$ in Bq kg^−1^. If the input is an elemental mass fraction rather than a radionuclide mass fraction, $x_{r}$ must include the appropriate isotopic abundance. Half-lives expressed in years must be converted to seconds before calculating activities in Bq. The uranium conversion below refers to ^238^U activity only and does not demonstrate equilibrium with ^226^Ra or other uranium-series daughters. For $x_{U}=10^{-6}$, $M_{238}=0.238\;\mathrm{kg}\;{\mathrm{mol}}^{-1}$ and $T_{1/2}=4.468\times 10^{9}$ a, the result is approximately $12.35\;\mathrm{Bq}\;{\mathrm{kg}}^{-1}$ for ^238^U. For $x_{Th}=10^{-6}$, $M_{232}=0.232\;\mathrm{kg}\;{\mathrm{mol}}^{-1}$ and $T_{1/2}=1.405\times 10^{10}$ a, the result is approximately $4.06\;\mathrm{Bq}\;{\mathrm{kg}}^{-1}$ for ^232^Th. For potassium, a 1% K concentration corresponds to 10 g of natural potassium per kg. The activity of ^40^K is calculated as

(A3) $${C_{40}}_{K}=x_{K}f_{40}\frac{N_{A}\lambda_{40}}{M_{K}},$$

where $f_{40}$ is the natural isotopic abundance of ^40^K. With $f_{40}≃1.17\times 10^{-4}$ and $T_{1/2}=1.248\times 10^{9}$ a, the result is approximately $313\;\mathrm{Bq}\;{\mathrm{kg}}^{-1}$ for ^40^K. These conversions are geochemical cross-checks and do not prove decay-chain equilibrium.

## Appendix B. Template for a Case-Study Results Table

**Table A1:** Recommended structure for future case-study reporting. Values shown as dashes indicate fields to be populated with measured data.

Sample/Fraction	$w_{i}$	^226^Ra	^232^Th	^40^K	^137^Cs	Notes
	(–)	$\mathrm{Bq}\;{\mathrm{kg}}^{-1}$	$\mathrm{Bq}\;{\mathrm{kg}}^{-1}$	$\mathrm{Bq}\;{\mathrm{kg}}^{-1}$	$\mathrm{Bq}\;{\mathrm{kg}}^{-1}$	
Bulk <2 mm	–	–	–	–	–	measured directly
<63μm	–	–	–	–	–	fine fraction
63–125 μm	–	–	–	–	–	fine sand
125–250 μm	–	–	–	–	–	fine to medium sand
250–500 μm	–	–	–	–	–	medium sand
500–1000 μm	–	–	–	–	–	coarse sand
1000–2000 μm	–	–	–	–	–	very coarse sand
Heavy-mineral concentrate	–	–	–	–	–	density/magnetic method specified
