# Supplementary material for: RadSed-INT: A Scenario-Aware Protocol for Radioactivity Assessment in Dynamic Beach Sediments
Source: Toxics. 2026 Jul 3;14(7):590. doi: 10.3390/toxics14070590 (PMC13417626; doi:10.3390/toxics14070590)
Supplement: Supplementary file 1 [file toxics-14-00590-s001.zip › toxics-4392362-supplementary_PROOFREAD.pdf]

Supplementary Material

RadSed-INT: A Scenario-Aware Protocol for Radioactivity Assessment in Dynamic Beach Sediments

Sebastiano Ettore Spoto, Roberta Somma and Antonio Trifirò

Purpose and link with the main manuscript

This Supplementary Material accompanies the main RadSed-INT manuscript. It is not a separate dataset and does not replace the core decision logic in the main text. Supplementary Table S1 expands the notation table used in the Methods, Supplementary Table S2 expands the scenario-centered regulatory translation matrix, Supplementary Table S3 expands the confounder checklist, Supplementary Table S4 lists HPGe correction and quality-assurance items, Supplementary Table S5 summarizes closure-uncertainty propagation schemes, Supplementary Table S6 provides the S4 artificial-radionuclide workflow and Supplementary Table S7 lists Tier 4/Tier 5 investigation triggers, Supplementary Table S8 provides standard grain-size equations, and Supplementary Table S9 gives a minimal worked example for radiometric mass closure. These tables are intended as implementation aids and should be updated when a case study is applied under a specific jurisdiction, beach setting or laboratory quality system. References cited in the Supplementary Material are also included in the main manuscript reference list.

Extended notation

Table S1. Extended notation used in RadSed-INT.

Symbol	Quantity	Use in the protocol
C_j	activity concentration	Dry-mass activity concentration of radionuclide or decay-chain segment j, in Bq kg^{-1}.
$\mathbf{x}_h$	horizontal position	Horizontal position on the beach surface; in transect designs it may be reduced to a cross-shore coordinate, with alongshore variability represented by replicated transects or explicit stratification.
N_ℓ^{net}	net peak area	Background-subtracted net peak area for gamma line ℓ used in HPGe activity estimation.
t_c, m_{dry}	live counting time and dry mass	Live counting time and dry sample mass used in the HPGe measurement model; counting time controls uncertainty and detection capability, not the physical activity concentration.
ε_ℓ, $P_{\gamma,\ell}$	efficiency and emission probability	Full-energy peak efficiency and photon emission probability per decay for gamma line ℓ.
$F_{q,\ell}$	correction factor	Multiplicative correction factor for an effect not already included in the net peak area or efficiency calibration.
$\mathbf{c}$, $\mathbf{V}$	line-estimate vector and covariance	Vector of line-specific activity estimates and corresponding covariance matrix used in multi-line averaging.
Q_j, χ^2_ν	line-consistency statistics	Covariant and diagonal diagnostic statistics for consistency among gamma-line estimates.

Continued on next page

Table S1. Extended notation used in RadSed-INT (continued).

Symbol	Quantity	Use in the protocol
$u(X)$, $u^2(X)$	standard uncertainty and variance	Standard uncertainty of X and its variance, i.e., the squared standard uncertainty used in uncertainty propagation.
$\mathcal{A}$	physical activity	Activity in Bq; used only where the physical decay rate of a quantity of material is needed.
$C_{A,j}$, $C_{B,j}$	A/B results	Activity concentration measured in the primary analytical sample A and retained confirmatory sample B.
w_i	grain-size mass fraction	Dry mass fraction of particle-size class i; $\sum_i w_i = 1$.
f_w	water fraction	Gravimetric water content or wet-mass fraction, kept distinct from w_i.
$F_{<63}$	fine fraction	Dry mass fraction below 63 μm; used as mud/fine-particle descriptor and possible carrier proxy.
f_{HM}	heavy-mineral fraction	Dry mass fraction of separated heavy minerals or heavy-mineral-rich material.
f_{CaCO_3}	carbonate fraction	Carbonate or bioclastic dilution proxy, usually expressed as a mass fraction.
z_{cs}	morphodynamic zone	Cross-shore or shoreface position used to separate sampling populations.
κ	magnetic susceptibility	Rapid proxy for Fe–Ti minerals and related heavy-mineral sorting where applicable.
D_{50}	median grain diameter	Sedimentological median grain size; not a dose quantity.
C_{bj}^{meas}, C_{bj}^{pred}	measured and predicted bulk activity	Bulk activity concentration measured directly and reconstructed from grain-size fractions.
EF_{ij}, RC_{ij}	enrichment and contribution	Fraction-specific enrichment factor and relative contribution to the reconstructed bulk activity.
ζ	depth coordinate	Vertical coordinate used for stratigraphic profiles, mini-cores and gamma attenuation with depth.
M_z, σ_I, Sk_I, K_G	grain-size statistics	Folk–Ward mean size, sorting, skewness and kurtosis.
ρ_s	matrix density	Density used for HPGe efficiency, attenuation or geometry transfer.
$\rho_d(\zeta)$	dry bulk density	Dry bulk density in a vertical profile as a function of depth ζ.
$\dot{D}_\gamma$	absorbed dose rate	Outdoor gamma absorbed dose rate in air, generally in nGy h^{-1}.
$E_{s,site}^{\text{ext}}$	site-related external dose	Annual external effective dose for occupancy scenario s calculated from the measured site sediment without background subtraction.
ΔE_s^{ext}	incremental external dose	Annual external effective dose above a declared local/regional background or pre-intervention baseline.
E_s^{tot}	total pathway dose	Sum of external and triggered internal pathway doses for scenario s, when those pathways are explicitly assessed.

Continued on next page

Table S1. Extended notation used in RadSed-INT (continued).

Symbol	Quantity	Use in the protocol
$E_{ref,s}$	dose reference for comparison	Declared reference, investigation or screening level used for a specified SDI form; not a mathematical default.
T_s	occupancy or scenario duration	Time basis for dose calculations; h a^{-1} for annual estimates or event duration when explicitly stated.
y^*, $y^\#$	characteristic limits	Decision threshold and detection limit in the radiometric characteristic-limits framework.
$SDI_{s,\mathrm{ext}}$, $SDI_{s,\mathrm{tot}}$	Sediment Dose Index forms	External-only and total-pathway ratios between a declared dose quantity and the selected reference or investigation level.
M_x	airborne mass concentration	Mass concentration of aerosol size fraction x in kg m^{-3}; x may denote inhalable, thoracic, respirable, particulate matter $(PM)_{10}$ or $PM_{2.5}$ material.
BR_s	breathing rate	Scenario-specific breathing rate in m^3 h^{-1} for inhalation-dose screening.
$C^{air}_{j,x}$	air activity concentration	Activity concentration of radionuclide j in airborne particle class x, in Bq m^{-3}.
$C_{x,j}$	dust-fraction activity concentration	Activity concentration of radionuclide j in aerosolizable or size-selected fraction x, in Bq kg^{-1}.
$\mathcal{I}_{j,res}$	resuspendable inventory	Resuspendable surface inventory of radionuclide j, commonly in Bq m^{-2}.
$C_{j,ing}$	ingestible-fraction activity	Activity concentration of radionuclide j in the sediment fraction relevant to incidental ingestion.
K_R	resuspension factor	Ratio between air activity concentration and resuspendable surface inventory; units are m^{-1} when inventory is in Bq m^{-2}.
$E^{\mathrm{inh}}_{s,ann}$	annual inhalation dose	Scenario-specific annual committed effective dose from inhalation of resuspended sediment particles when T_s is annual occupancy.
$e^{inh}_{j,x}$	inhalation dose coefficient	Committed effective dose per unit intake for radionuclide j and particle/absorption class x, in Sv Bq^{-1}.
M^{ing}_s	ingested sediment mass	Scenario-specific annual mass of incidentally ingested sediment, in kg a^{-1}.
E^{ing}_s	ingestion effective dose	Scenario-specific committed effective dose from incidental ingestion of sediment.
$E^{\mathrm{Rn/Tn}}_s$	radon/thoron pathway dose	Scenario-specific dose term used only when radon/thoron exhalation or inhalation is explicitly triggered and quantified.
e^{ing}_j	ingestion dose coefficient	Committed effective dose per unit intake by ingestion for radionuclide j, in Sv Bq^{-1}.
A_{eff}	effective specific activity	Retained only where it is the conventional notation of a national construction-material index.

Extended regulatory translation matrix

Table S2. Extended illustrative, non-binding, non-exhaustive and date-sensitive regulatory translation matrix. The table is a navigation aid only and must be updated against current primary legislation before application.

Jurisdiction	Main instruments or concepts	Relevant quantities	Protocol interpretation
European Union	Basic Safety Standards and building-material provisions [Council of the European Union, 2013]; RP112 and International Atomic Energy Agency (IAEA) guidance [European Commission, 1999, International Atomic Energy Agency, 2023a].	Public dose reference level; activity concentration index $I = C_{Ra}/300 + C_{Th}/200 + C_K/3000$ for building materials.	S1 requires dose-based beach assessment. I is applied only if the sediment becomes or enters a building/construction material.
Italy / EU example	D.Lgs. 101/2020 transposes the European Basic Safety Standards (BSS); NORM industries and clearance/exemption are treated in a dose and activity-concentration framework [Repubblica Italiana, 2020, Trevisi et al., 2023].	NORM exemption/clearance and public/worker dose criteria; building-material index when relevant.	Useful European case study because it separates NORM work activities, residues and construction products from natural outdoor beaches.
Germany	Radiation protection framework for building products and natural radionuclides; German Federal Office for Radiation Protection (BfS) guidance on building-material radionuclides [Bundesamt für Strahlenschutz, 2024].	Activities of ^{226}Ra, ^{232}Th and ^{40}K; activity concentration index; additional gamma dose from construction products.	Strong European example for S3. Not a direct S1 beach criterion.

Continued on next page

Table S2. Extended illustrative regulatory translation matrix (continued).

Jurisdiction	Main instruments or concepts	Relevant quantities	Protocol interpretation
United States	U.S. Nuclear Regulatory Commission (NRC) public-dose and license-termination criteria; U.S. Environmental Protection Agency (EPA) TENORM and Superfund risk-based tools [United States Nuclear Regulatory Commission, 2026, United States Environmental Protection Agency, 2025, 2026].	Public dose limit for licensed operations; 25 mrem a^{-1} criterion for unrestricted release of licensed sites; TENORM definition; risk-based preliminary remediation goals (PRGs).	S1 uses background and dose context. S2/S4 require state/federal pathway-specific interpretation. No generic federal beach-specific NORM concentration limit is used here as a universal screening value.
Canada	Canadian NORM Guidelines and public dose concepts [Health Canada, 2011].	0.3 mSv a^{-1} first investigation level for public exposure; 1 mSv a^{-1} public dose limit in radiation protection contexts.	Good example of a tiered screening approach for NORM. Applicable to S2/S3 and to conservative S1 screening.
Australia	Australian Radiation Protection and Nuclear Safety Agency (ARPANSA) Radiation Protection Series (RPS) 15 and graded NORM approach [Australian Radiation Protection and Nuclear Safety Agency, 2008].	Optimization and dose-based management; public exposure around 1 mSv a^{-1} as a reference context.	Especially relevant for mineral-sand provinces where heavy-mineral enrichment and industrial handling coexist.
Japan	Nuclear Regulation Authority (NRA) guideline for raw materials and products containing U or Th [Nuclear Regulation Authority of Japan, 2009]; separate post-accident frameworks for artificial radionuclides.	U/Th raw material and product criteria; artificial radiocesium criteria in post-accident contexts.	NORM and post-accident radiocesium must be treated as separate modules. Do not transfer accident-derived waste criteria to natural beaches.

Continued on next page

Table S2. Extended illustrative regulatory translation matrix (continued).

Jurisdiction	Main instruments or concepts	Relevant quantities	Protocol interpretation
China	GB 6566 for radionuclides in building materials [Standardization Administration of China, 2010]; IAEA summaries of building-material control [International Atomic Energy Agency, 2023a].	Internal and external exposure indices for building materials; ^{226}Ra, ^{232}Th and ^{40}K.	Primarily S3. For S1, use dose-based beach assessment and in situ measurements.
Russia	Effective specific activity for construction materials, e.g. $A_{eff} = A_{Ra} + 1.3A_{Th} + 0.09A_K$ in reported Russian practice [Sidelnikova et al., 2016].	Construction-material classes, commonly including 370, 740 and 1500 Bq kg^{-1} effective-activity thresholds.	Useful alternative index-based system for S3. It should not be used as a universal criterion for beach sediment left in situ.
No beach-specific national criteria	International Atomic Energy Agency (IAEA) and International Commission on Radiological Protection (ICRP) principles of justification, optimization, reference levels and dose constraints [International Atomic Energy Agency, 2014, 2023b, International Commission on Radiological Protection, 2007, 1999].	Dose-based tiers; NORM screening at order-of-magnitude levels for planned-exposure control.	Apply the default tiered protocol defined in the main manuscript; document that tiers are screening criteria, not national law.

Extended confounder checklist

Table S3. Extended environmental, geomorphic and methodological confounder checklist.

Factor	Effect on activity or dose-rate interpretation	Protocol response
Rainfall and wet deposition	Temporary gamma increase from deposited radon progeny; increased moisture attenuates terrestrial gamma photons and changes density.	Avoid routine in situ surveys during or soon after rain; record rainfall for at least 72 h; if rainy-condition dose is relevant, treat it as a separate scenario.
Sediment moisture and pore water	Does not change dry-mass activity, but affects in situ dose rate, sample mass, density and gamma attenuation.	Report wet and dry mass, moisture and density; use dry-mass activities; apply geometry and self-attenuation corrections.
Wave climate and swash sorting	Size-density sorting can concentrate zircon, monazite, rutile, ilmenite and magnetite in thin layers or strandlines.	Record wave height, period and direction; repeat after storms; fractionate by size and density; do contact gamma scanning.
Tides and beach-face position	The active swash zone migrates during the tidal cycle; intertidal sediment may be recently reworked.	Sample within a defined tidal window; record tidal stage and morphological zone; compare equivalent zones through time.
Storms and overwash	Storm erosion can remove light minerals, expose lag deposits or bury enriched layers during recovery.	Include event-based pre/post-storm transects where feasible; distinguish transient veneer from persistent deposit using depth intervals.
Buried enriched layers	A high-activity unit below the present surface may contribute weakly to routine surface samples but become exposure-relevant after erosion, scraping or excavation.	Activate Tier 4 vertical mini-cores; report vertical ratios, activity-depth centroids and uncertainty-tested stratification.
Wind and aeolian winnowing	Dry backshore and dunes can accumulate heavy minerals or remove fines.	Record wind conditions and dune/backshore morphology; include dry beach and dune/berm zones where relevant.

Continued on next page

Table S3. Extended environmental, geomorphic and methodological confounder checklist (continued).

Factor	Effect on activity or dose-rate interpretation	Protocol response
Dust resuspension and aerosolizable fines	Fine, dry or mechanically disturbed sediment can generate inhalable, thoracic or respirable particles; dust composition may differ from bulk composition and create an inhalation pathway not represented by external gamma indices.	Activate Tier 5 only where aerosolization is plausible; measure fine/dust fraction activity, moisture, handling or wind conditions, particle-size convention, and airborne mass or dustiness.
River discharge and floods	New sediment pulses may change mineralogy, grain size, ^{137}Cs, ^{210}Pb and natural radionuclide signatures.	Treat river-influenced sectors as separate strata; record discharge/flood history; include upstream/provenance samples where possible.
Beach nourishment, dredging and grooming	Imported or mechanically mixed sand can reset the radiogeochemical baseline and destroy natural layering.	Record management history; sample before and after operations; treat nourished sectors separately from natural reference sectors.
Shells, carbonates and organic matter	Carbonates and shells may dilute U–Th-bearing heavy minerals; organic matter and Fe–Mn phases can bind surface-reactive radionuclides.	Report carbonate/shell fraction and loss-on-ignition where relevant; do not remove bioclasts unless the measurand is redefined.
Thin black-sand laminae	Semi-infinite homogeneous dose models may over- or under-estimate exposure if activity is concentrated in a veneer.	Use contact and 1 m measurements; micro-sample laminae separately; compare field dose with HPGe-derived dose.
Artificial radionuclides	^{137}Cs, ^{134}Cs, ^{60}Co or ^{241}Am may indicate fallout, accident influence or site contamination rather than NORM.	Apply artificial-radionuclide decision criteria; interpret in a separate regulatory module.
Detector geometry and background	Detector height, surface roughness, cosmic contribution and calibration can alter in situ dose rates.	Standardize height and integration time; measure local background; document calibration and uncertainty.
Radon/thoron exhalation	Exhalation may increase where Ra/Th-bearing minerals are present, but it is strongly controlled by moisture, porosity, permeability, tides, wind and chamber sealing.	Treat RAD7-type continuous alpha-spectrometric monitor measurements, or equivalent radon/thoron measurements, as ancillary pathway indicators; report chamber geometry, humidity control, background, integration time and flux model.

Continued on next page

Table S3. Extended environmental, geomorphic and methodological confounder checklist (continued).

Factor	Effect on activity or dose-rate interpretation	Protocol response
Sample processing	Loss of fines, rinsing, poor splitting and geometry mismatch can bias bulk–fraction closure.	Use mass recovery, dry-mass control, sealed geometries, replicate splits and the mass-closure statistic defined in the main manuscript.
A/B confirmatory sampling	A single sample can be analytically precise but non-confirmable if no retained confirmatory sample exists.	Archive the appropriate B state for anomalies and management decisions; evaluate duplicates with relative percent difference (RPD) and Z_{AB}.

HPGe correction and quality-assurance checklist

Table S4. Minimum HPGe correction and quality-assurance items for sediment matrices and fractions.

Correction / issue	Minimum implementation requirement	Reporting item
Efficiency transfer	Use a calibrated geometry or a validated efficiency-transfer method for the counted matrix, density and fill height. Quartz-rich, carbonate-rich and heavy-mineral-rich fractions should not be assumed equivalent unless density and attenuation effects are negligible.	Geometry, fill height, density, calibration source or transfer method, and uncertainty component.
Self-attenuation	Apply transmission, matrix-matched, Monte Carlo or validated empirical correction where photon attenuation differs from the calibration matrix.	Energy-specific attenuation correction and uncertainty.
^{210}Pb at 46.5 keV	Use matrix-specific self-absorption correction or equivalent matrix-matched calibration; build a thickness–density correction curve when density/fill height varies across fractions.	Correction curve, matrix range, calibration standard and acceptance criterion.
Coincidence summing	Evaluate only when source–detector distance, detector efficiency and cascade structure make summing relevant. Avoid close geometries for high-efficiency counting unless corrected.	Geometry, summing assessment/correction and whether the effect is included in efficiency calibration.
High count rate, dead time and pile-up	Control count-rate effects through geometry, reduced mass, increased source–detector distance, dilution or validated detector settings; shortening live time alone does not remove dead time or pile-up.	Dead-time range, pile-up check, geometry/dilution decision and recount criterion.
Line consistency	Compare multiple lines using the covariance-aware estimator in the main text where possible.	Line list, estimated activity per line, covariance assumptions and Q_j or diagonal diagnostic.

Continued on next page

Table S4. Minimum HPGe correction and quality-assurance items for sediment matrices and fractions (continued).

Correction / issue	Minimum implementation requirement	Reporting item
Chain disequilibrium	Interpret gamma lines as decay-chain segments unless equilibrium is demonstrated.	Proxy lines, sealing date, ingrowth time, container type, and equilibrium acceptance criterion.

Closure uncertainty propagation schemes

Table S5. Practical uncertainty propagation schemes for bulk–fraction closure.

Scheme	When applicable	Treatment in RadSed-INT
Independent-input scheme	Bulk and fractions are prepared, counted and calibrated independently, and mass-fraction errors are treated as independent approximations.	Use the diagonal form of $u^2(C_{bj}^{pred})$ and omit the covariance term in Z_j only after this assumption is stated.
Shared-parameter scheme	Bulk and fractions share calibration curves, nuclear data, density correction, dry-mass normalization, sieving recovery or the compositional constraint $\sum_i w_i = 1$.	Use $u^2(C_{bj}^{pred}) = (\nabla C)^T \Sigma_x (\nabla C)$ and retain $-2\,\mathrm{cov}(C_{bj}^{meas}, C_{bj}^{pred})$ in the closure statistic.
Bias check when covariance is ignored	Covariances are suspected but not quantifiable.	Report the diagonal Z_j as a screening diagnostic, state that covariance may change its magnitude, and avoid regulatory conclusions based only on marginal failure of closure.

S4 artificial-radionuclide operation tree

Table S6. Trigger-based operation tree for the S4 artificial-radionuclide module.

S4 branch	Typical trigger	Minimum response
Fallout or accident gamma emitters	^{137}Cs, ^{134}Cs, ^{60}Co or ^{241}Am above decision criteria or site-specific background.	Keep the artificial inventory separate from NORM dose indices; confirm line identity, map spatial pattern, and select a radionuclide- and context-specific regulatory pathway.

Table S6. Trigger-based operation tree for the S4 artificial-radionuclide module (continued).

S4 branch	Typical trigger	Minimum response
Discrete hot particles	Local contact anomaly, line inconsistency, or high count rate not represented by homogenized bulk.	Conduct particle screening, autoradiography or micro-sampling; assess contact/skin dose if local beta/gamma emission may dominate.
Non-gamma or weak-gamma nuclides	Suspected ^{90}Sr, Pu isotopes, ^{210}Po or other nuclides not resolved by routine HPGe.	Use radiochemical separation, beta counting, alpha spectrometry or mass-spectrometric methods as appropriate; do not infer absence from a gamma-only spectrum.
Industrial TENORM particles	Discrete mineral-processing residues, slag, scale, or anthropogenic black particles.	Treat as S4/S2 hybrid; document morphology, mineralogy and radionuclide suite; avoid classification by natural Ra–Th–K indices alone.

Tier 4 and Tier 5 investigation triggers

Table S7. Illustrative trigger indicators for Tier 4 vertical radiostratigraphy and Tier 5 aeolian/inhalation assessment. These are protocol triggers, not regulatory limits.

Tier	Trigger indicator	Interpretation and action
Tier 4	$VR_{j,k} > 2$ with an uncertainty-resolved contrast.	Moderate buried/surface enrichment trigger; repeat or confirm the depth interval and report uncertainty.
Tier 4	$VR_{j,k} > 5$, maximum activity below 5 cm, or activity centroid within the expected storm/grooming erosion depth.	Strong vertical investigation trigger; evaluate exposure after erosion, scraping, excavation or beach recovery.
Tier 4	Field–laboratory mismatch between contact dose, 1 m dose and 0–5 cm HPGe result.	Investigate thin surface veneers, buried laminae, hot particles, moisture/attenuation effects or unrepresented material.
Tier 5	Visible dust, dry erodible surface, stockpile, grooming, scraping, vehicle traffic or documented dust-generating operations.	Characterize airborne mass or dustiness; separate natural wind deflation from human disturbance.
Tier 5	Aerosolizable or size-selected fraction enriched relative to bulk, for example $EF_x \geq 2$ as a screening indicator.	Measure activity in the relevant particle-size convention; do not substitute $< 63\,\mu$m sediment for PM_{10} without validation.

Table S7. Illustrative trigger indicators for Tier 4 vertical radiostratigraphy and Tier 5 aeolian/inhalation assessment (continued).

Tier	Trigger indicator	Interpretation and action
Tier 5	Annual or event-based dust duration available from wind records or maintenance logs.	Use source-specific durations in $E^{inh}_{s,wind}$ and $E^{inh}_{s,work}$; report whether the dose is event-based or annualized.

Standard grain-size equations used as sedimentological covariates

Table S8. Standard sedimentological equations used by RadSed-INT as explanatory covariates. These formulas are not new contributions of the protocol; they are provided here to make the sedimentological variables reproducible.

Quantity	Equation	Use in RadSed-INT
Krumbein phi scale	$\phi = -\log_2\left(\frac{d_g}{1\ \text{mm}}\right)$	Converts grain diameter to the logarithmic sedimentological scale.
Folk–Ward mean size	$M_z = \frac{\phi_{16} + \phi_{50} + \phi_{84}}{3}$	Central grain-size descriptor used as a physical covariate.
Inclusive graphic sorting	$\sigma_I = \frac{\phi_{84} - \phi_{16}}{4} + \frac{\phi_{95} - \phi_5}{6.6}$	Describes hydraulic selection, mixing and potential concentration of dense minerals.
Inclusive graphic skewness	$Sk_I = \frac{\phi_{16} + \phi_{84} - 2\phi_{50}}{2(\phi_{84} - \phi_{16})} + \frac{\phi_5 + \phi_{95} - 2\phi_{50}}{2(\phi_{95} - \phi_5)}$	Identifies fine or coarse tails, veneers, lag deposits and mixed populations.
Graphic kurtosis	$K_G = \frac{\phi_{95} - \phi_5}{2.44(\phi_{75} - \phi_{25})}$	Describes concentration of the grain-size distribution and tail behavior.

Minimal worked example for radiometric mass closure

Table S9. Synthetic worked example showing how a bulk activity concentration can be reconstructed from grain-size fractions. The numbers are illustrative and do not represent a field dataset.

Fraction	w_i	$u(w_i)$	C_{ij} (Bq kg^{-1})	Contribution w_iC_{ij}
$< 63\ \mu$m	0.10	0.005	220 ± 20	22.0
63–125 μm	0.20	0.008	120 ± 10	24.0
125–250 μm	0.35	0.010	90 ± 8	31.5
250–500 μm	0.25	0.009	70 ± 7	17.5

Table S9. Synthetic worked example showing bulk–fraction closure (continued).

Fraction	w_i	$u(w_i)$	C_{ij} (Bq kg^{-1})	Contribution w_iC_{ij}
500–2000 μm	0.10	0.005	50 ± 6	5.0
Mass-weighted prediction	$\sum_i w_i = 1.00$	–	–	$C_{bj}^{pred} = 100.0$
Measured bulk	–	–	$C_{bj}^{meas} = 101 \pm 6$	–
Closure diagnostic	–	–	–	$Z_j = (101 - 100)/\sqrt{6^2 + 4.8^2} = 0.13$

For this illustrative example, the diagonal approximation gives $u(C_{bj}^{pred}) \approx 4.8$ Bq kg^{-1} and the closure statistic is far below a typical warning threshold such as $|Z_j| = 2$. In a real application, shared calibration, density, dry-mass normalization and compositional constraints may introduce covariances; in that case, the covariance-aware form of the closure statistic in the main manuscript should be used. A simple machine-readable CSV template and Python script are provided as Supplementary Files S1 and S2 for transparent implementation of the same calculation.

References

Australian Radiation Protection and Nuclear Safety Agency. Safety guide: Management of naturally occurring radioactive material (norm). radiation protection series no. 15. ARPANSA, Yallambie, 2008. URL `https://www.arpansa.gov.au/sites/default/files/legacy/pubs/rps/rps15.pdf`. Available online: `https://www.arpansa.gov.au/sites/default/files/legacy/pubs/rps/rps15.pdf` (accessed on 19 May 2026).

Bundesamt für Strahlenschutz. Natural radionuclides in building materials. Federal Office for Radiation Protection, Germany, 2024. URL `https://www.bfs.de/EN/topics/ion/environment/building-materials/radionuclides/radionuclides.html`. Available online: `https://www.bfs.de/EN/topics/ion/environment/building-materials/radionuclides/radionuclides.html` (accessed on 19 May 2026).

Council of the European Union. Council directive 2013/59/euratom of 5 december 2013 laying down basic safety standards for protection against the dangers arising from exposure to ionising radiation. Official Journal of the European Union L 13, 17 January 2014, 2013. URL `https://eur-lex.europa.eu/legal-content/EN/TXT/?uri=CELEX:32013L0059`. Available online: `https://eur-lex.europa.eu/legal-content/EN/TXT/?uri=CELEX:32013L0059` (accessed on 19 May 2026).

European Commission. Radiological protection principles concerning the natural radioactivity of building materials. radiation protection 112. Directorate-General for Energy, 1999. URL `https://energy.ec.europa.eu/publications/radiological-protection-principles-concerning-natural-radioactivity-building-materials-rp-112_en`. Available online: `https://energy.ec.europa.eu/publications/radiological-protection-principles-concerning-natural-radioactivity-building-materials-rp-112_en` (accessed on 19 May 2026).

Health Canada. Canadian guidelines for the management of naturally occurring radioactive materials (norm). Prepared by the Canadian NORM Working Group of the Federal Provincial Territorial

Radiation Protection Committee, 2011. URL https://www.canada.ca/en/health-canada/services/publications/health-risks-safety/canadian-guidelines-management-naturally-occurring-radioactive-materials.html. Available online: https://www.canada.ca/en/health-canada/services/publications/health-risks-safety/canadian-guidelines-management-naturally-occurring-radioactive-materials.html (accessed on 19 May 2026).

International Atomic Energy Agency. Radiation protection and safety of radiation sources: International basic safety standards. general safety requirements part 3. IAEA Safety Standards Series No. GSR Part 3, International Atomic Energy Agency, Vienna, 2014. URL https://www-pub.iaea.org/MTCD/Publications/PDF/Pub1578_web-57265295.pdf. Available online: https://www-pub.iaea.org/MTCD/Publications/PDF/Pub1578_web-57265295.pdf (accessed on 19 May 2026).

International Atomic Energy Agency. Regulatory control of exposure due to radionuclides in building and construction materials. safety reports series no. 117. International Atomic Energy Agency, Vienna, 2023a. URL https://www-pub.iaea.org/MTCD/Publications/PDF/PUB1992_web.pdf. Available online: https://www-pub.iaea.org/MTCD/Publications/PDF/PUB1992_web.pdf (accessed on 19 May 2026).

International Atomic Energy Agency. Application of the concept of exemption. general safety guide no. gsg-17. International Atomic Energy Agency, Vienna, 2023b. URL https://www-pub.iaea.org/MTCD/Publications/PDF/PUB2060_web.pdf. Available online: https://www-pub.iaea.org/MTCD/Publications/PDF/PUB2060_web.pdf (accessed on 19 May 2026).

International Commission on Radiological Protection. Protection of the public in situations of prolonged radiation exposure. ICRP Publication 82, Annals of the ICRP 29(1–2), 1999.

International Commission on Radiological Protection. The 2007 recommendations of the international commission on radiological protection. ICRP Publication 103, Annals of the ICRP 37(2–4), 2007.

Nuclear Regulation Authority of Japan. Guideline for ensuring safety of raw materials and products containing uranium or thorium. Former MEXT guideline, Japan, 2009. URL https://www.nra.go.jp/data/000034146.pdf. Available online: https://www.nra.go.jp/data/000034146.pdf (accessed on 19 May 2026).

Repubblica Italiana. Decreto legislativo 31 luglio 2020, n. 101. attuazione della direttiva 2013/59/euratom, che stabilisce norme fondamentali di sicurezza relative alla protezione contro i pericoli derivanti dall'esposizione alle radiazioni ionizzanti. Gazzetta Ufficiale della Repubblica Italiana, Rome, 2020. URL https://www.normattiva.it/uri-res/N2Ls?urn:nir:stato:decreto.legislativo:2020;101!vig=. Available online: https://www.normattiva.it/uri-res/N2Ls?urn:nir:stato:decreto.legislativo:2020;101!vig= (accessed on 28 May 2026).

O. P. Sidelnikova, L. I. Khorzova, and P. A. Sidyakin. Radiation-related hygienic assessment of construction materials in urbanized complexes in the volgograd region. *Spatium*, 36:46–54, 2016. doi: 10.2298/SPAT1636046S.

Standardization Administration of China. Gb 6566-2010: Limits of radionuclides in building materials. National Standard of the People's Republic of China, 2010. URL https://openstd.samr.gov.cn/bzgk/std/newGbInfo?hcno=254CCB9E2333A1803914C70A5B2EBECB. Available online: https://openstd.samr.gov.cn/bzgk/std/newGbInfo?hcno=254CCB9E2333A1803914C70A5B2EBECB (accessed on 19 May 2026).

R. Trevisi, M. Ampollini, A. Bogi, S. Bucci, E. Caldognetto, G. La Verde, F. Leonardi, L. Luzzi, C. Nuccetelli, I. Peroni, F. Picciolo, G. Pratesi, F. Trotti, R. Ugolini, G. Venoso, and M. Pugliese. Radiological protection in industries involving norm: A (graded) methodological approach to characterize the exposure situations. *Atmosphere*, 14(4):635, 2023. doi: 10.3390/atmos14040635.

United States Environmental Protection Agency. Technologically enhanced naturally occurring radioactive materials (tenorm). EPA radiation protection information, 2025. URL https://www.epa.gov/radiation/technologically-enhanced-naturally-occurring-radioactive-materials-tenorm. Available online: https://www.epa.gov/radiation/technologically-enhanced-naturally-occurring-radioactive-materials-tenorm (accessed on 19 May 2026).

United States Environmental Protection Agency. Radionuclide preliminary remediation goals for superfund. EPA PRG calculator and user guidance, 2026. URL https://epa-prgs.ornl.gov/radionuclides/. Available online: https://epa-prgs.ornl.gov/radionuclides/ (accessed on 19 May 2026).

United States Nuclear Regulatory Commission. 10 cfr part 20 – standards for protection against radiation. Electronic Code of Federal Regulations, 2026. URL https://www.ecfr.gov/current/title-10/chapter-I/part-20. Available online: https://www.ecfr.gov/current/title-10/chapter-I/part-20 (accessed on 19 May 2026).
