# Supplementary figures and images for: RadSed-INT: A Scenario-Aware Protocol for Radioactivity Assessment in Dynamic Beach Sediments
Source: Toxics. 2026 Jul 3;14(7):590. doi: 10.3390/toxics14070590 (PMC13417626; doi:10.3390/toxics14070590)

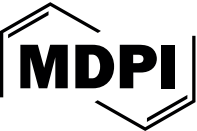

Supplement: Supplementary file 1 [file toxics-14-00590-s001.zip › Definitions/logo-mdpi-eps-converted-to.pdf]

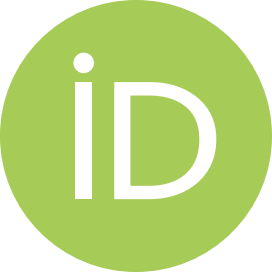

Supplement: Supplementary file 1 [file toxics-14-00590-s001.zip › Definitions/logo-orcid.pdf]

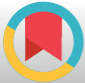

Check for updates

Supplement: Supplementary file 1 [file toxics-14-00590-s001.zip › Definitions/logo-updates-eps-converted-to.pdf]
